# High HIV and active tuberculosis prevalence and increased mortality risk in adults with symptoms of TB: a systematic review and meta‐analyses

**DOI:** 10.1002/jia2.25162

**Published:** 2018-07-31

**Authors:** Marriott Nliwasa, Peter MacPherson, Ankur Gupta‐Wright, Mphatso Mwapasa, Katherine Horton, Jon Ø Odland, Clare Flach, Elizabeth L. Corbett

**Affiliations:** ^1^ Helse Nord Tuberculosis Initiative Department of Pathology College of Medicine Blantyre Malawi; ^2^ Malawi‐Liverpool‐Welcome Trust Clinical Research Programme Blantyre Malawi; ^3^ Clinical Research Department London School of Hygiene & Tropical Medicine (LSHTM) London UK; ^4^ Department of Clinical Sciences Liverpool School of Tropical Medicine Liverpool UK; ^5^ Department of Community Medicine Faculty of Health Sciences UiT The Arctic University of Norway Tromsø Norway; ^6^ School of Public Health University of Pretoria Pretoria South Africa; ^7^ Department of Primary Care & Public Health Sciences King's College London London UK

**Keywords:** Tuberculosis, HIV, screening, mortality, policy, health systems

## Abstract

**Introduction:**

HIV and tuberculosis (TB) remain leading causes of preventable death in low‐ and middle‐income countries (LMICs). The World Health Organization (WHO) recommends HIV testing for all individuals with TB symptoms, but implementation has been suboptimal. We conducted a systematic literature review and meta‐analyses to estimate HIV and TB prevalence, and short‐term (two to six months) mortality, among adults with TB symptoms at community‐ and facility level.

**Methods:**

We searched Embase, Global Health and MEDLINE databases, and reviewed conference abstracts for studies reporting simultaneous HIV and TB screening of adults in LMICs published between January 2003 and December 2017. Meta‐analyses were performed to estimate prevalence of HIV, undiagnosed TB and mortality risk at different health system levels.

**Results:**

Sixty‐two studies including 260,792 symptomatic adults were identified, mostly from Africa and Asia. Median HIV prevalence was 19.2% (IQR: 8.3% to 40.4%) at community level, 55.7% (IQR: 20.9% to 71.2%) at primary care level and 80.7% (IQR: 73.8% to 84.6%) at hospital level. Median TB prevalence was 6.9% (IQR: 3.3% to 8.4%) at community, 20.5% (IQR: 11.7% to 46.4%) at primary care and 36.4% (IQR: 22.9% to 40.9%) at hospital level. Median short‐term mortality was 22.6% (IQR: 15.6% to 27.7%) among inpatients, 3.1% (IQR: 1.2% to 4.2%) at primary care and 1.6% (95% CI: 0.45 to 4.13, n = 1 study) at community level.

**Conclusions:**

Adults with TB symptoms have extremely high prevalence of HIV infection, even when identified through community surveys. TB prevalence and mortality increased substantially at primary care and inpatient level respectively. Strategies to expand symptom‐based TB screening combined with HIV and TB testing for all symptomatic individuals should be of the highest priority for both disease programmes in LMICs with generalized HIV epidemics. Interventions to reduce short‐term mortality are urgently needed.

## 1 Introduction

Tuberculosis (TB) and human immunodeficiency virus (HIV) are the two leading causes of adult infectious disease deaths worldwide. In 2016, there were 1.7 million deaths due to TB globally, including 0.4 million deaths among HIV‐positive individuals 1. Reducing the high mortality from HIV‐related TB has become an increasingly high priority of The Joint United Nations Programme on HIV/AIDS (UNAIDS) and The United States President's Emergency Plan for AIDS Relief (PEPFAR) programmes, and the recently ratified End TB Strategy includes a 35% reduction in TB deaths by 2025 among other targets 2.

Early diagnosis and treatment are key components of both HIV and TB programmes 3. HIV testing is being scaled‐up as countries work towards the UNAIDS “90‐90‐90” HIV diagnosis and care targets for 2020 4. Similarly, the End TB Strategy places increased emphasis on systematic screening for TB, including facility attendees and high risk communities, as part of early TB diagnosis 4. Guidelines for TB/HIV collaborative activities released by the World Health Organization (WHO) in 2004 were updated in 2012 5. The 2004 guidelines focused on HIV testing and care for diagnosed TB patients and TB screening and prevention as part of HIV care, with annual reporting requirements. The 2012 guidelines recommend HIV testing among patients with suspected TB, noting high HIV prevalence and suboptimal service integration for this group, but without good quality of evidence, additional data reporting recommendations, or consideration of HIV testing in the context of TB screening programmes 5.

TB patients undergoing treatment in national TB programmes are at high risk of death, especially if HIV positive 6, 7. A 2011 meta‐analysis estimated that 18.8% (95% confidence interval [CI]: 14.8% to 22.8%) of HIV‐positive and 3.5% (95% CI: 2.0% to 4.9%) of HIV‐negative TB patients die during TB treatment 7, 8. Adults with symptoms such as cough with or without confirmed TB disease in high HIV prevalence settings also face a high risk of early mortality, with HIV infection a risk factor for worse outcomes 9. Despite the 2012 recommendation and suggestions of similar HIV prevalence and short‐term mortality risks as notified TB patients, routine management of adults with TB symptoms in health services remains suboptimal with missed opportunities for HIV testing and referral for ART 9. In addition, monitoring for HIV testing and linkage to ART is still not well established among patients with TB symptoms, with neither HIV nor TB programmes reporting coverage or outcomes. Accurate estimates of HIV and TB burden and risk of death among adults with symptoms of TB may help policymakers, researchers and implementers to prioritize appropriate collaborative interventions at the different levels of the healthcare system. We, therefore, set out to systematically summarize HIV prevalence, active TB prevalence and mortality risk among people with symptoms of TB (with or without confirmed TB disease) identified at community level (the general population) and in health facilities in low‐ and middle‐income countries (LMICs) with different underlying burden of HIV and TB.

## 2 Methods

### 2.1 Search strategy

In accordance with our published protocol (PROSPERO ID: CRD42015021944), we searched MEDLINE, Embase and Global Health electronic databases using a predefined search strategy (Table S1) for studies reporting outcomes of HIV and TB screening among adults identified in the community or during health facility attendance in LMICs that were published between 1 January 2003 and 31 December 2017. The start of the literature search was set in 2003 because it is the year the ART scale‐up commenced in many LMICs. We additionally hand‐searched abstracts from the Union World Conferences on Lung Health and International AIDS Society (IAS) Conferences from January 2014 to December 2017.

### 2.2 Eligibility criteria

Studies were eligible for inclusion if they offered participants systematic screening for HIV at the time of TB screening. Acceptable TB screening algorithms comprising either symptom screening followed by microbiological confirmatory testing for active TB, or universal microbiological testing for TB, as well as HIV testing. We included randomized controlled trials (RCTs), cohort studies, cross‐sectional studies, TB prevalence surveys, studies of evaluation of new TB diagnostic tests and published reports of programmatic activities, but excluded commentaries, editorials, case reports, case series, economic analyses and qualitative studies.

Studies conducted in countries defined to be low‐ or middle income by the World Bank lending groups in 2016 were included. We included studies that recruited only adults (≥16 years), or where both children and adults were included and age‐disaggregated data were reported. We excluded studies that reported only a preselected, unrepresentative group of participants, including diagnosed TB or HIV‐positive patients only, sputum smear‐negative patients, TB household contacts, participants with suspected multidrug‐resistant TB, miners and pregnant women.

Studies were imported into an EndNote X7 database and duplicates were removed. MN screened the titles and abstracts of all studies against inclusion and exclusion criteria, and the full text of potentially eligible studies were reviewed in duplicate by MN and AGW against inclusion and exclusion criteria using a predesigned electronic assessment form. Discrepancies were resolved by discussion between the reviewers, with arbitration by a third reviewer (PM) in case of disagreement.

### 2.3 Data extraction

MN and AGW extracted data from selected studies using a previously piloted electronic data extraction form; inconsistencies were resolved by discussion. For each study, we extracted the author name, year, country and setting, and we described details of the TB and HIV screening algorithms used. The following data were extracted for main outcomes: total number of participants, number screened for TB symptoms and number screening positive for TB symptoms. For adults with TB symptoms, we then extracted the numbers screened and diagnosed with HIV and TB and the number of deaths.

### 2.4 Assessment of study quality

For assessment of methodological quality, RCTs were distinguished from non‐randomized studies (see Supplementary material). For RCTs, the Cochrane Collaboration's Tool for Assessing Risk of Bias was used. For non‐randomized studies, a modified version of the Newcastle‐Ottawa Scale was used to assess selection of participants and methods of assessment of each of the three outcomes (Table S2). For the HIV prevalence outcome, we assessed uptake of HIV testing and the quality of the diagnostic algorithm used; studies relying only on participants’ verbal report were classified as having high risk of bias. Similarly, for the TB prevalence outcome, we assessed uptake of testing and if TB disease was bacteriologically confirmed (i.e. based on sputum smear microscopy, Xpert^®^ MTB/RIF or culture testing) or clinically diagnosed (when classified as TB patient without bacteriological confirmation). For mortality risk, we assessed methods of ascertainment of deaths (i.e. hospital/study record, verbal autopsy or vital registration) and the completeness of follow‐up of the cohorts. For each study, the overall risk of bias for each outcome was categorized as low‐, high‐ or unclear depending on the assessment of the domains above.

### 2.5 Definitions

We classified participants symptomatic of TB as either having chronic cough (defined as ≥2 weeks as commonly used in community surveys) or as having ≥1 symptom in the WHO‐recommended four‐symptom screening tool (current cough of any duration, fever, night sweats, or weight loss).

We defined four levels of healthcare. The community level encompassed the general population that is studies recruiting adults from households or temporary mobile service in residential areas (excluding those in schools, prisons or other institutions). The primary care level included general practitioner services and health centres. The hospital‐level category included studies on inpatients admitted to a ward and stayed at least one night in hospital. An additional category of mixed setting was used to define studies that included participants from more than one level that is both primary care clinics and hospitals (without disaggregated data), or for participants recruited at specialist outpatient clinics.

For each study, we defined national adult (aged 15 to 49 years old) HIV prevalence by year and country using UNAIDS estimates. For studies spanning more than one year, estimates were based on the middle year, if study covered an even number of years the average national prevalence of the two middle years was used. National incidence of TB was estimated from data reported in the WHO global TB reports for each year, and categorized as: low (<30 per 100,000), moderate (30 to 100 per 100,000), medium (100 to 300 per 100,000) and high (>300 per 100,000) 10. Geographical distribution of studies was categorized based on WHO regions 11. For mortality outcome, *short‐term* or *early* mortality was defined as deaths occurring in the first six months of follow‐up among those with TB symptoms.

A case of TB disease diagnosed after recruitment was defined as report by a study of a bacteriologically confirmed case (at least one positive sputum smear microscopy sample, positive culture for *Mycobacterium tuberculosis* (MTB), or a positive Xpert^®^ MTB/RIF result) or a clinically diagnosed case (when TB treatment was initiated without bacteriological confirmation).

### 2.6 Statistical analyses

The primary outcomes of the study were: HIV prevalence (the number of participants with confirmed HIV infection divided by the total number with TB symptoms), prevalence of active TB (the number with active TB divided by the number with TB symptoms) and mortality risk (the number of participants confirmed to have died by six months divided by the number with TB symptoms). We stratified analyses by level of healthcare (community, primary care, mixed and hospital inpatients). For HIV and TB prevalence outcomes, we undertook subgroup analyses by geographical region, type of TB symptoms, national HIV prevalence and TB incidence. For HIV, we also determined the number needed to screen (NNS) to detect a newly diagnosed HIV‐positive individual. The NNS for TB was not conducted because it was discussed in detail in a 2013 systematic literature review by Shapiro *et al*. 12.

We assessed heterogeneity between studies using the *I* ^2^ statistic. Meta‐analyses were conducted using random effects models to estimate weighted summary outcome measures and 95% CI for each of the three outcomes, stratified by level of healthcare. Arcsine transformation of proportions was implemented in the calculation of pooled prevalence to handle zero or unitary values. When it was not appropriate to conduct meta‐analyses (i.e. if substantial heterogeneity with an *I* ^2^ ≥ 50%) 13, prevalence estimates were summarized as medians and interquartile ranges (IQR).

Meta‐regression analyses were performed to examine associations between HIV and TB prevalence and geographical region, group of TB symptoms, country‐level HIV prevalence and country‐level TB incidence. Characteristics showing strong association with respective outcomes on univariate meta‐regression were included in the multivariate meta‐regression. The variable TB symptom type (chronic cough only or ≥1 symptom from the WHO tool) was included in the model *a priori*. Due to the small number of studies reporting mortality, meta‐regression analyses were not conducted for this outcome. Analyses were conducted using R version 3.2.3 (The R Foundation for Statistical Computing, Vienna, 2016).

### 2.7 Ethics statement

This review used published data and ethical review was not required.

## 3 Results

### 3.1 Characteristics of included studies

**Figure 1:**
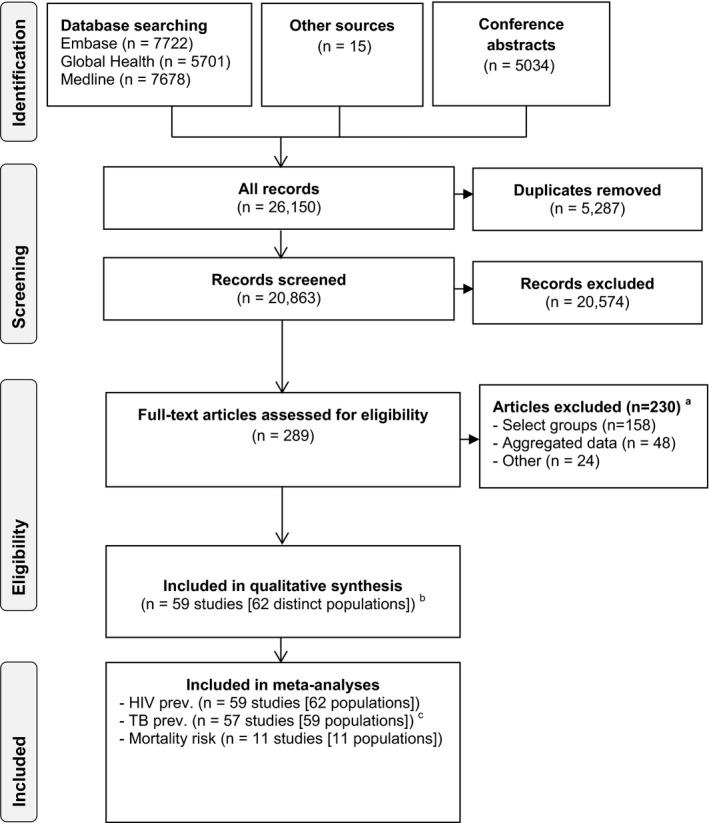
PRISMA flow diagram for process of selecting included studies. **(a)** Reasons for exclusion are explained in supplementary material. **(b)** One study, Boehme *et al*., was a multisite study (six sites), four sites were eligible; the total number of individual studies was 62. **(c)** Two studies did not report on results of TB testing.

The search strategy identified 20,863 records, from which we selected 289 eligible manuscripts (Figure 1). We included 59 manuscripts in the qualitative synthesis; 58 manuscripts reported single‐site studies and one manuscript reported on a multisite study (four eligible sites were treated as individual studies). The final number of studies included in the analysis was 62; all 62 for HIV prevalence outcome, 59 for TB prevalence and 11 for mortality risk (Figure 1). Common reasons for exclusion included studies in preselected, unrepresentative populations (68.7%, 158/230) and not presenting outcome data stratified by presence of TB symptoms (20.9%, 48/230) ( Table S3).

In keeping with our requirement for systematic HIV testing in parallel with TB investigations, most studies were from the African region (51/62, 82.3%), with the largest number from South Africa (13/62, 21.0%) (Table 1). In total, studies included 260,792 adults with TB symptoms, with one study from India 14 contributing 115,308 participants.

**Table 1:** Characteristics of included studies

Region	Countries	Studies	Level of care				Number with TB symptoms
			Community	Primary care	Hospital inpatients	Mixed	
African region							
Southern Africa^a^	5	27	4	15	6	2	37,285
East Africa^b^	5	17	5	5	2	5	27,019
West Africa^c^	3	6	1	1	1	3	4,775
Central Africa^d^	1	1	0	1	0	0	49,832
SE Asia region^e^	2	8	0	4	0	4	122,237
W Pacific region	1	1	1	0	0	0	12,201
Americas	2	2	1	0	0	1	7,443
Total	19	62	12	26	9	15	260,792

a Countries are South Africa (studies=13), Botswana (2), Malawi (5), Zambia (4) and Zimbabwe (3).

b Countries are Ethiopia (5), Kenya (2), Tanzania (3), Uganda (6) and Rwanda (1).

c Countries are Guinea Bissau (2), Ghana (1), Nigeria (3).

d One study from Democratic Republic of Congo.

e Countries are Thailand (3) and India (5), includes one large study from India with 115,308 participants.

SE Asia, South East Asia; W Pacific, Western Pacific.

Twelve (12/62, 19.4%) studies were conducted at community level, 26/62 (41.9%) at primary care level, 9/62 (14.5%) at hospital level (inpatients) and 15/62 (24.2%) were conducted in mixed settings (Table 1). Studies at community level were cross‐sectional 15, 16, 17, 18, 19 and cohort in design (Table 2) 20, 21, 22. Studies at primary care level were either diagnostic evaluations 23, 24, 25, 26, 27, 28, 29, 30, programme evaluations 31, 32, 33 or other cross‐sectional designs 34, 35, 36, 37. Studies among hospital inpatients were predominantly diagnostic evaluations 38, 39, 40, 41.

**Table 2:** TB diagnostic procedures of included studies

Country, author, year^a^	Study design	Study description	Participant Eligibility criteria	TB diagnosis algorithm	Participants with symptoms	HIV prev.	TB prev.
					n	n (%)	n (%)
Community level							
Cambodia, Lorent (2012) 18	Cross‐sectional study	Active TB case finding (door to door strategy)	Any TB symptoms	Microscopy (fluorescence), Xpert MTB/RIF, culture (LJ) + species ID and DST	12,201	319 (2.6)	774 (6.3)
Ethiopia, Deribew (2012) 17	Cross‐sectional study	Regional TB prevalence survey	Cough ≥2 weeks	Microscopy (fluorescence, ZN), culture (LJ) + species ID	482	5 (0.9)	17 (2.9)
Guinea Bissau, Bjerregaard‐Andersen (2009) 22	Cross‐sectional study	Regional TB prevalence survey	Cough or any two other TB symptoms	ZN microscopy, CXR	116	24 (20.7)	8 (6.9)
Malawi, Nliwasa (2016) 20	Cohort study (individuals with chronic cough vs no cough)	Assessing TB yield and mortality risk	Cough ≥2 weeks	Microscopy (fluorescence), Xpert MTB/RIF, culture (MGIT) + species ID	178	56 (31.5)	6 (3.4)
Haiti, Rivera (2017) 65	Cross‐sectional study	Active TB case finding (door to door strategy)	Cough ≥2 weeks	CXR, Microscopy, Xpert MTB/RIF,	5598	528 (9.4)	1,000 (17.9)
Rwanda, 2014 49	Cross‐sectional study	National TB prevalence survey	Cough (any duration) or abnormal CXR	Microscopy (fluorescence), culture (MGIT) + species ID	4747	218 (4.6)	54 (1.1)
South Africa, Kranzer (2012) 19	Cross‐sectional study	Mobile multi‐disease screening service	Any TB symptoms, or if HIV positive or diabetic	Microscopy (fluorescence), culture (MGIT) + species ID	1385	758 (54.7)	103 (7.4)
Tanzania, 2013 42	Cross‐sectional study	National TB prevalence survey	Cough ≥2 weeks or abnormal CXR	Microscopy (fluorescence), culture (MGIT) + species ID	6271	782 (12.5)	149 (2.4)
Uganda, Sekandi (2014) 21	Cross‐sectional study	Active TB case finding (door to door strategy)	Cough ≥2 weeks	ZN microscopy and culture (LJ)	199	82 (41.2)	39 (19.6)
Uganda, 2017 66	Cross‐sectional study	National TB prevalence survey	Cough ≥2 weeks or abnormal CXR	ZN microscopy, Xpert MTB/RIF and culture (LJ).	4386	417 (9.5)	160 (3.6)
Zambia, Ayles (2009) 15	Cross‐sectional study	TB prevalence survey – selected area	All adults in rural and urban communities	ZN microscopy, culture (MGIT & LJ) + species ID	578	230 (39.8)	34 (5.9)
Zimbabwe, Corbett (2010) 16	Cross‐sectional study	TB prevalence survey – selected area	All adults from randomly selected households	Microscopy (fluorescence), culture (LJ) + species ID	333	153 (45.9)	37 (11.1)

Country, author, year^a^	Study design	Study description	Participant eligibility criteria	TB diagnosis	No. with symptoms	HIV prev.	TB prev.
					n	n (%)	n (%)
Primary care							
DR Congo, Yotebieng (2013) 67	Cross‐sectional study	Evaluation of HIV PITC in patients with TB symptoms	Cough any duration or other symptoms	Not described	28,568	3,029 (10.6)	‐
Ethiopia, Deribew (2010) 34	Cross‐sectional study	Evaluation of HIV PITC in patients with TB symptoms	Chronic cough or other symptoms	Not described	506	81 (16.0)	233 (46.0)
Ethiopia, Sahle (2017) 68	Cross‐sectional study	LAM evaluation study	Any TB symptom	Urinary LAM, LJ culture	122	21 (17.2)	35 (28.7)
Guinea‐Bissau, Rudolf (2017) 69	Prospective observational study	Assessing biomarkers for predicting mortality	Cough any duration or other symptoms	Not described	1011	161 (15.9)	101 (10.0)
India, Boehme (2011) 23	Cross‐sectional study	Xpert MTB/RIF evaluation study	Chronic cough or suspected MDR TB	Xpert MTB/RIF, culture (LJ, MGIT, Ogawa) + species ID and DST	902	40 (4.4)	108 (12.0)
India, Achanta (2012) 32	Cross‐sectional study	Evaluation of HIV PITC in patients with TB symptoms	Cough ≥2 weeks or other symptoms	Smear microscopy	2,918	246 (8.4)	407 (13.9)
India, Naik (2012) 33	Cross‐sectional study	Evaluation of HIV PITC in patients with TB symptoms	Clinician identified	Not described	1539	108 (7.0)	100 (6.5)
India, Kumar (2016) 14	Cross‐sectional study	Evaluation of HIV PITC in patients with TB symptoms	Patients submitting sputum samples	Not described	115,308	7,559 (6.5)	‐
Kenya, Kivihya‐Ndugga (2003) 70	Cross‐sectional study	Assessing TB diagnostic algorithms	Clinician identified	Microscopy (ZN, fluorescence) and culture (LJ)	993	128 (12.9)	554 (55.8)
Kenya, Odhiambo, (2008) 35	Cross‐sectional study	Evaluation of HIV PITC in patients with TB symptoms	Any TB symptom	smear microscopy	5457	2,988 (54.8)	2,595 (47.6)
Malawi, Munthali (2006)^71^	Cross‐sectional study	Assessing HIV prevalence in patients with TB symptoms	Chronic cough	Not described	145	79 (54.5)	31 (21.4)
Malawi, Nliwasa (2016) 29	Cross‐sectional study	TB‐LAMP diagnostic evaluation study	Cough ≥2 weeks	Microscopy (fluorescence, ZN), LAMP, Xpert MTB/RIF, culture (MGIT) + species ID	273	121 (44.3)	56 (20.5)

Country, author, year^a^	Study design	Study description	Participant eligibility criteria	TB diagnosis	No. with symptoms	HIV prev.	TB prev.
					n	n (%)	n (%)
South Africa, Mwansa‐Kambafwile (2011)	Cross‐sectional study	Xpert MTB/RIF evaluation study	Clinician identified	Microscopy, Xpert MTB/RIF	1981	1442 (72.8)	271 (18.8)
South Africa, Brunet (2011) 72	Cross‐sectional study	Assessing HIV and smoking prevalence in patients with TB symptoms	Clinician identified	Microscopy and culture	424	119 (28.1)	286 (67.5)
South Africa, Scott (2011) 30	Cross‐sectional study	Xpert MTB/RIF evaluation study	Clinician identified	MTBDRplus and the LCTB assays, Xpert MTB/RIF	319	220 (69.0)	175 (54.9)
South Africa, Theron (2011) 73	Cross‐sectional study	Xpert MTB/RIF evaluation study	Definition: clinician identified	Microscopy (fluorescence), Xpert MTB/RIF, culture (MGIT) and DST	480	130 (27.1)	232 (48.3)
South Africa, Hanrahan (2013) 27	Prospective observational study	Xpert MTB/RIF evaluation study	Cough any duration or other	Smear microscopy, culture and Xpert MTB/RIF	641	443 (69.1)	116 (18.1)
South Africa, Cox (2014)^25^	Pragmatic Randomized Trial	Xpert MTB/RIF evaluation study	Cough any duration or other symptoms	Xpert MTB/RIF, smear microscopy, culture, DST	1985	965 (48.6)	424 (21.4)
South Africa, Geldenhuys (2014) 74	Cross‐sectional study	Assessing sputum collection methods	Chronic cough or other symptoms	Culture used (MGIT) + species ID and DST	555	118 (21.3)	105 (18.9)
South Africa, Van Rie (2014)^37^	Cross‐sectional study	Assessing uptake of TB screening	Prolonged cough and weight loss	Xpert MTB/RIF	1505	933 (62.0)	90 (6.0)
South Africa, Churchyard (2015)^24^	Cluster‐randomized trial	Xpert MTB/RIF evaluation study	Clinician identified	Microscopy and Xpert MTB/RIF	4656	2,206 (47.4)	385 (8.3)
South Africa, Hanrahan (2015)^26^	Prospective observational study	Xpert MTB/RIF evaluation study	Cough any duration or other	Smear microscopy, culture and Xpert MTB/RIF	1861	1,336 (71.8)	204 (11.0)
Uganda, Srikantiah (2007)^36^	Cross‐sectional study	Assessing HIV prevalence in patients with TB symptoms	Clinician identified	ZN microscopy plus CXR	565	238 (42.1)	378 (66.9)
Zambia, Muyoyeta (2015) 28	Cross‐sectional study	Xpert MTB/RIF evaluation study	Cough any duration	Xpert MTB/RIF, chest X‐ray, fluorescence microscopy	13,926	7,190 (52.6)	2,861 (20.5)
Zimbabwe, Dlodlo (2015)^50^	Cross‐sectional study	Evaluation of HIV PITC in patients with TB symptoms	Cough ≥ 2 weeks	Microscopy, Xpert MTB/RIF, if HIV positive	422	297 (70.4)	‐
Zimbabwe, Munyati (2004) 75	Cross‐sectional study	Assess causes of chronic cough	Chronic cough	Microscopy (ZN) and culture (LJ) + species ID	544	454 (83.5)	184 (33.8)

Country, author, year^a^	Study design	Study description	Participant eligibility criteria	TB diagnosis	No. with symptoms	HIV prev.	TB prev.
					n	n (%)	n (%)
Mixed settings							
Ethiopia, Legesse (2010) 76	Cross‐sectional study	Diagnostic evaluation study	Clinician identified	Microscopy (ZN) and culture (LJ)	140	27 (19.3)	37 (26.4)
Ethiopia, Belay (2015) 77	Cross‐sectional study	Assessing HIV prevalence in patients with TB symptoms	Chronic cough	Microscopy (ZN) and culture (LJ)	325	82 (34.9)	110 (33.8)
Ghana, Adjei (2006) 78	Cross‐sectional study	Assessing HIV prevalence in patients with TB symptoms	Clinician identified	Microscopy (ZN)	277	128 (46.2)	108 (39.0)
India, Kaur (2011) 79	Cross‐sectional study	Assessing HIV prevalence in patients with TB symptoms	Clinician identified	Sputum smear microscopy, culture	618	3 (0.5)	243 (39.3)
Malawi, Van Lettow (2015) 44	Prospective observational study	Assessing 6 m outcomes for inpatients	Definition: adults in chronic cough register. Tests: microscopy and chest X‐ray	Routine care HIV testing	348	191 (54.9)	53 (15.2)
Peru, Boehme (2011) 23	Cross‐sectional study	Xpert MTB/RIF evaluation study	Chronic cough or suspected MDR TB	Xpert MTB/RIF, culture and DST	1845	5 (0.3)	209 (11.3)
Nigeria, Aliyu (2013)^80^	Cross‐sectional study	Assessing prevalence of NTM	Any TB symptom	Culture (MGIT, LJ) and species ID	1603	378 (23.6)	444 (27.7)
Nigeria, Chuks (2013) 81	Cross‐sectional study	Assessing HIV prevalence in patients with TB symptoms	Patients submitting sputum	microscopy (ZN)	1544	184 (11.9)	237 (15.3)
South Africa, Boehme (2011) 23	Cross‐sectional study	Xpert MTB/RIF evaluation study	Chronic cough or suspected MDR TB	Xpert MTB/RIF, culture and DST	2522	947 (37.5)	493 (19.5)
Tanzania, Rachow (2011) 82	Cross‐sectional study	Diagnostic evaluation study of Xpert	Patients with suspected TB	Microscopy (ZN), Xpert MTB/RIF and culture/DST	292	172 (58.9)	146 (50.0)
Tanzania, Mulder (2017) 45	Cross‐sectional study	Assessing use of rats in TB diagnosis	Patients with suspected TB	Xpert MTB/RIF and Culture	771	264 (34.2)	345 (44.7)
Thailand, Kawkitinarong (2017) 83	Cross‐sectional study	Diagnostic evaluation study of Xpert	Patients with suspected TB	Microscopy, Xpert MTB/RIF and culture	494	128 (25.9)	355 (71.9)
Thailand, Nanta (2011) 84	Cross‐sectional study	Diagnostic evaluation study of Xpert	No description	ICT‐TB tests	401	206 (51.4)	146 (36.4)
Thailand, Pinyopornpanish (2015) 85	Cross‐sectional study	Diagnostic evaluation study of Xpert	Patients with suspected TB	Microscopy, Xpert MTB/RIF culture	57	15 (26.3)	27 (47.4)
Uganda, Boehme (2011) 23	Cross‐sectional study	Xpert MTB/RIF evaluation study	Chronic cough or suspected MDR TB	Xpert MTB/RIF, culture/DST	372	254 (68.3)	147 (39.5)

Country, author, year^a^	Study design	Study description	Participant Eligibility criteria	TB diagnosis	No. with symptoms	HIV prev.	TB prev.
					n	n (%)	n (%)
Hospital inpatients							
Botswana, Talbot (2004) 86	Cross‐sectional study	Assessing HIV prevalence in patients with TB symptoms	Cough ≥2 weeks	Microscopy (ZN), culture (LJ, MGIT, blood) + 4 serological TB tests	465	384 (82.6)	175 (37.6)
Botswana, Morse (2008) 47	Cross‐sectional study	Assessing PTB diagnosis from different samples	Clinician identified	fluorescent microscopy and culture (LJ, MGIT)	140	113 (80.7)	57 (40.7)
Malawi, Gawa (2011) 43	Cross‐sectional study	TB programme evaluation study	Clinician identified	Smear microscopy and chest X‐ray	141	50 (35.5)	11 (7.8)
Nigeria, Hirao (2007) 87	Cross‐sectional study	Assessing number of samples for TB diagnosis	Cough ≥3 weeks	Smear microscopy and culture	224	106 (47.3)	78 (34.8)
South Africa, Shah (2009) 88	Cross‐sectional study	LAM diagnostic evaluation study	Patients with suspected TB	Fluorescent microscopy, LAM, culture (MGIT, MycoF Lytic) + species ID	499	422 (84.6)	282 (56.5)
Uganda, Yoon (2012) 41	Prospective observational study	Evaluation study of Xpert MTB/RIF	Cough for ≥2 weeks	Florescence microscopy, Xpert MTB/RIF, culture (LJ, MGIT) + species ID	477	362 (75.9)	262 (54.9)
Uganda, Jones‐Lopez (2014) 89	Cross‐sectional study	Assessing small membrane filtration for TB diagnosis	Cough ≥2 weeks or other	Fluorescence microscopy, Xpert MTB/RIF, culture (MGIT, LJ) + species ID	212	173 (81.6)	70 (33.0)
Zambia, O'Grady (2012) 39	Cross‐sectional study	Evaluation study of Xpert MTB/RIF	Patients submitting sputum	fluorescent smear microscopy, Xpert MTB/RIF, culture (MGIT) + species ID	881	595 (67.5)	202 (22.9)
Zambia, Bates (2013) 38	Cross‐sectional study	Evaluation study of Xpert MTB/RIF	Productive cough	Microscopy (fluorescence), culture (MGIT) and DST	98	62 (63.3)	26 (26.5)

a Sorted by country alphabetical order, for each level of healthcare.

TB, tuberculosis; LJ, Lowenstein‐Jensen media; ID, Identification; ZN, Ziehl‐Neelsen stain; MGIT, mycobacteria growth indicator tube; DST, drug susceptibility testing; ICT, immunochromatographic tests; PITC, provider‐initiated testing and counselling; NTM, non‐tuberculous mycobacteria.

### 3.2 Quality of included studies

For HIV prevalence outcome, 47/62 (75.8%) of the studies had low risk of bias while 15/62 (24.2%) had high or unclear risk of bias (Supplementary material). Studies were categorized as having high risk of bias for the HIV prevalence due to poor determination of HIV status (6/62, 9.7%)^22^, ^23^, ^24^, ^32^, ^38^, ^39^; unclear or low uptake of HIV testing (8/62, 12.9%)^18^, ^23^, ^34^, ^42^, ^43^, ^44^, ^45^; and not describing how HIV status was determined (1/62, 1.6%) 38. Most (53/59, 89.8%) studies had low risk of bias for the estimation of TB prevalence. There were only 11/62 (17.7%) studies that reported on the outcome of mortality; 63.6% (7/11) of them had high risk of bias. Reasons for the high risk of bias were high loss to follow‐up rates, (4/11, 36.4%) 20, 25, 27, 30, and not describing how vital status was ascertained, (3/11, 27.3%) 40, 46, 47.

### 3.3 Prevalence of HIV by level of health care

Of the total 260,792 adults with symptoms of TB reported in 62 included studies, 184,601 (70.8%) were successfully screened for HIV. There was substantial variability in estimated HIV prevalence (range: 0.5% to 100%) (Table 2 and Figure S1). By level of care, the median HIV prevalence among adults with TB symptoms was 19.2% (IQR: 8.3% to 40.4%, n = 12 studies) at community level, 55.7% (IQR: 20.9% to 71.2%, n = 26 studies) at primary care level, 28.6% (IQR: 21.4% to 52.0%, n = 15 studies) in mixed settings, and was 80.7% (IQR: 73.8% to 84.6%, n = 9 studies) among hospital inpatients (Table 3).

**Table 3:** Random‐effects meta‐regression for HIV prevalence in adults with TB symptoms

	Studies	Participants screened	Median HIV prevalence	Univariate meta‐regression	*P*	Multivariate meta‐regression	*P*
			% (IQR)	Prevalence ratio (95% CI)		Prevalence ratio (95% CI)	
Level of care							
Community	12	32,472	19.2 (8.3 to 40.4)	1		1	
Primary care	26	139,933	55.7 (20.9 to 71.2)	1.34 (1.11 to 1.61)	0.002	1.32 (1.15 to 1.50)	<0.001
Mixed	15	9230	28.6 (21.4 to 52.0)	1.14 (0.93 to 1.40)	0.216	1.29 (1.12 to 1.50)	<0.001
Hospital inpatients	9	2966	80.7 (73.8 to 84.6)	1.90 (1.50 to 2.40)	<0.001	1.66 (1.40 to 1.97)	<0.001
National HIV prevalence							
Low (0% to 5%)	25	134,965	17.2 (9.4 to 26.3)	1		1	
High (>5%)	37	49,636	62.3 (45.9 to 73.8)	1.56 (1.37 to 1.76)	<0.001	1.45 (1.30 to 1.62)	<0.001
Group of symptoms							
Any TB symptom	41	170,885	32.8 (17.2 to 59.4)	1		1	
Chronic cough	21	13,716	68.3 (42.1 to 80.1)	1.24 (1.05 to 1.46)	0.011	1.14 (1.03 to 1.27)	0.013
WHO region^a^							
Non‐Africa region	11	84,658	9.9 (5.1 to 26.1)	1			
Africa region	51	99,943	54.5 (26.1 to 70.9)	1.42 (1.16 to 1.72)	<0.001		

a There was collinearity between geographical region and country‐level HIV prevalence, geographical region was not included in the multivariate analysis.

In univariate and multivariate meta‐regression, HIV prevalence among adults with TB symptoms was significantly higher in the following studies: among hospital inpatients, from the African region, with high national HIV prevalence, and those reporting chronic cough only (Table 3). Compared to adults with TB symptoms in the community, those at higher levels of care had higher HIV prevalence; adjusted prevalence ratio (aPR) 1.32 (95% CI: 1.15 to 1.50) at primary care, aPR 1.29 (95% CI: 1.12 to 1.50) in mixed settings and aPR 1.66 (95% CI: 1.40 to 1.97) among hospital inpatients. Adults with TB symptoms from countries with higher HIV prevalence also had higher HIV prevalence, aPR 1.45 (95% CI: 1.30 to 1.62).

Seven studies reported on the number of participants newly diagnosed with HIV following screening (Table S6). At community level, the number of adults with TB symptoms needed to screen to detect one new HIV‐positive individual was 11 in Malawi 48, 4 in South Africa 19 and 121 in Rwanda 49. In primary care clinics the NNS was 2 in Malawi 29 and Zimbabwe 50, 4 in Zambia 51 and 30 in India (Table S6) 32.

### 3.4 Prevalence of TB disease by level of healthcare

There were 59 studies (155,167 adults with TB symptoms) that reported on results of TB screening. In these studies, estimated TB prevalence ranged from 0.8% to 71.9% (Table 2). By level of healthcare, the median TB prevalence was lowest at community level (6.9% [IQR: 3.3% to 8.4%, n = 12 studies]), followed by primary care level (20.5% [IQR: 11.5% to 46.8%, n = 23 studies]), mixed settings (36.4% [IQR: 22.9% to 41.0%, n = 15 studies]) and hospital inpatients (34.8 [IQR:26.5% to 40.7%, n = 9 studies]) (Table 4).

**Table 4:** Random‐effects meta‐regression for TB prevalence in adults with TB symptoms

	Studies	Participants screened	Median TB prevalence	Univariate meta‐regression	*p*	Multivariate meta‐regression	*p*
			% (IQR)	Prevalence ratio (95% CI)		Prevalence ratio (95% CI)	
Level of care							
Community	12	35,187	6.9 (3.3 to 8.4)	1			
Primary care	23	105,234	20.5 (11.5 to 46.8)	1.29 (1.13 to 1.49)	<0.001	1.27 (1.11 to 1.46)	<0.001
Mixed	15	11,609	36.4 (22.9 to 41.0)	1.42 (1.22 to 1.66)	<0.001	1.42 (1.22 to 1.65)	<0.001
Hospital inpatients	9	3,137	34.8 (26.5 to 40.7)	1.43 (1.21 to 1.71)	<0.001	1.45 (1.22 to 1.72)	<0.001
National HIV prevalence							
Low (0% to 5%)	24	99,334	23.1 (9.3 to 37.1)	1			
High (>5%)	35	55,833	24.1 (11.0 to 44.1)	1.05 (0.93 to 1.19)	0.414		
National TB incidence							
Moderate/medium incidence	28	99,014	20.3 (9.1 to 39.1)	1			
High incidence	15	36,819	26.5 (17.9 to 41.2)	1.08 (0.94 to 1.26)	0.270		
Very high incidence	16	19,334	18.8 (10.3 to 42.6)	1.04 (0.90 to 1.21)	0.562		
Symptom group							
Any TB symptom	39	138,490	26.4 (9.1 to 46.7)	1		1	
Chronic cough	20	16,677	19.9 (11.3 to 33.8)	0.94 (0.83 to 1.07)	0.355	0.94 (0.84 to 1.03)	0.289
WHO region							
Non‐Africa region	11	67,647	13.9 (9.4 to 37.9)	1			
Africa region	48	87,520	22.2 (10.7 to 39.8)	1.05 (0.90 to 1.23)	0.524		

On univariate analysis, neither high national HIV prevalence (PR 1.05 [95% CI: 0.93 to 1.19]) nor high national TB incidence (PR 1.08 [95% CI: 0.94 to 1.26]) was associated with higher TB prevalence among adults with TB symptoms (Table 4), although statistical power was limited by the small number of studies from low HIV prevalence settings. In addition, there was no association with geographical region or group of symptoms used (Table 4).

**Figure 2:**
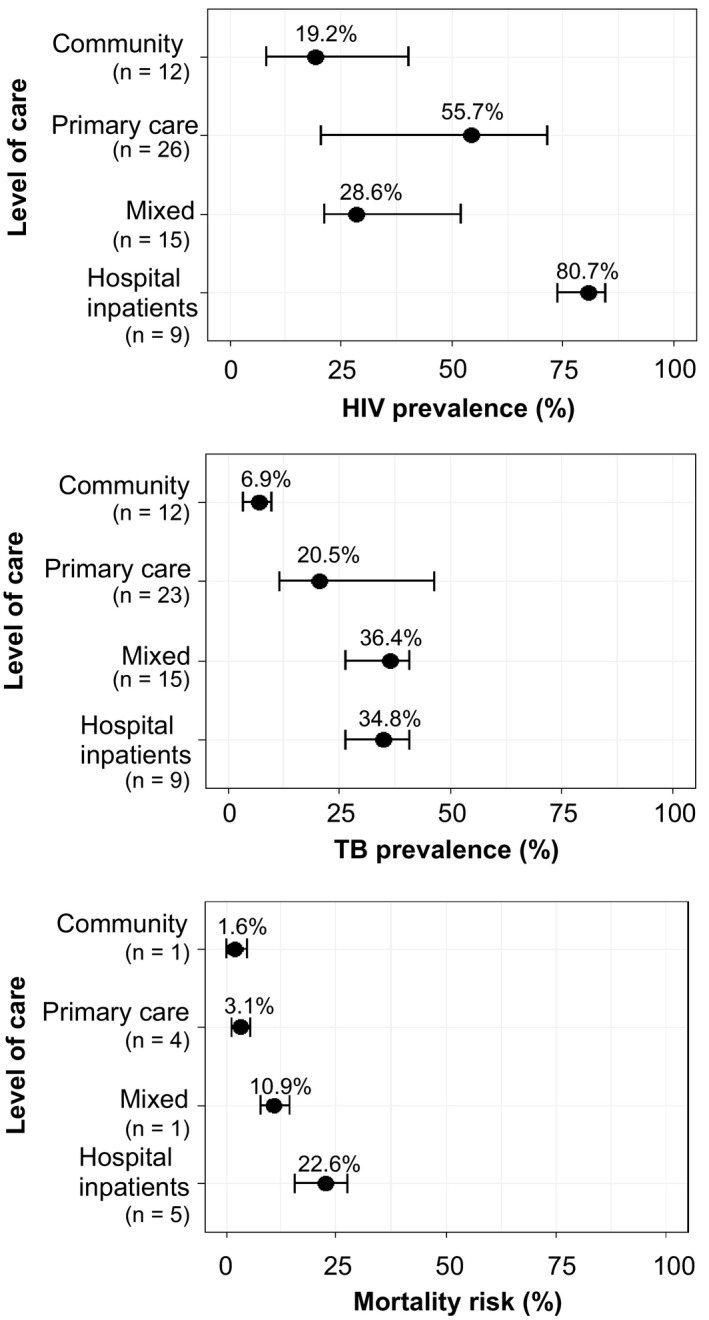
Summary HIV prevalence, TB prevalence and mortality risk at six months.

On univariate and multivariate analysis, TB prevalence was higher in symptomatic adults identified in all of primary care setting (aPR 1.27 [95% CI: 1.11 to 1.46]), mixed settings (aPR 1.44 [95% CI: 1.21 to 1.72]) and among hospital inpatients (aPR 1.42 [95% CI: 1.21 to 1.64]) than those in the community (Table 4). However, the 95% CI for TB prevalence ratios overlapped at levels higher than community, therefore differences were not statistically significant (also see Figure 2).

### 3.5 Mortality in adults with symptoms of tuberculosis

Eleven studies were included for analysis of cumulative incidence (risk) of mortality reported between two and six months of follow‐up; all were from Africa (Figure S2). Given the small number of studies we did not attempt to differentiate mortality by follow‐up duration, instead considering all as short‐term mortality (i.e. up to six months). Short‐term mortality risk was highest among hospital inpatients, with a median risk of death of 22.6% (IQR: 15.6% to 27.7%, n = 3 studies). Median risk of short‐term death was substantially lower among participants identified at primary care level (3.1% [IQR: 1.2% to 4.2%, n = 6 studies]) and community (1.6% [95% CI: 0.45 to 4.13], n = 1 study).

### 3.6 Influence of study quality on estimates

There was no significant difference in the estimate of HIV prevalence between low‐quality studies (median 34.0% [IQR: 19.2% to 68.1%]) and high‐quality studies (median 46.2% [IQR: 20.3% to 69.7%]), prevalence ratio 0.98 (95% CI: 0.81 to 1.19) (Table S7). Similarly, there was also no difference in estimate of TB prevalence between low‐quality studies (median 15.4% [IQR: 8.9% to 28.9%]) and high‐quality studies 22.1% (IQR: 11.1% to 39.8%), PR 0.89 (95% CI: 0.74 to 1.08) (Table S7). Notably, estimates of mortality risk were mostly based on low‐quality studies 54.5% (6/11), representing the need for better quality studies for this outcome.

## 4 Discussion

The main findings of this study, largely restricted to settings with generalized HIV epidemics by our selection criteria of studies reporting systematic HIV testing during investigation of TB symptoms, demonstrate extremely high HIV prevalence even for patients identified in the community during TB prevalence surveys (median 19.2% HIV prevalence). HIV prevalence was higher than TB prevalence in patients with TB symptoms at every level in the health system. The prevalence of previously undiagnosed TB was also substantial, increasing from 6.9% of adults with TB symptoms identified in the community to 20.5% and 34.8% in primary care facilities and inpatient units respectively. Patients admitted to hospital with TB symptoms had a very high risk of short‐term mortality (median 22.6%). These findings emphasize the critical need for adults identified with TB symptoms in HIV prevalent settings to be prioritized for HIV testing and ART services, including adults identified during community outreach or prevalence surveys. There is also urgent need to better understand and intervene to reduce short‐term mortality in this patient group, most pressingly so for inpatients, although mortality at primary care level (3.1% after two to six months follow‐up) was also disturbingly high.

Adults with TB symptoms identified in communities, primary care settings and in hospitals had median HIV prevalence of 19.2%, 55.7% and 80.7% respectively (Figure 2). National HIV prevalence was independently associated with higher HIV prevalence among symptomatic adults in meta‐regression. In Malawi and South Africa, countries with very high national HIV prevalence, the numbers of adults with TB symptoms needed to screen (NNS) at community level to detect one new HIV‐positive patient were low at 11 and 4 respectively 19, 48. In Malawi, the NNS from a single primary care study was 2, but the current National Strategic Plan for HIV/AIDS does not prioritize outpatients with TB symptoms for expanded provider‐initiated HIV testing and counselling (Table 5) 52. Instead HIV programmes have continued to focus on established target groups such as young and pregnant women, missing the opportunity to combine forces with TB programmes to provide an essential service for this high HIV prevalence patient group.

**Table 5:** Recommendations and future directions

Identified problem	Research directions	Programme priorities	References
Community level			
High HIV prevalence among adults with cough	Cluster randomized controlled trials to increase access to HIV testing in the community	Implement initiatives to achieve UNAIDS 90‐90‐90 targets: Community strategies, e.g. home‐based or mobile HIV testing and HIV self‐testing (HIVST)	55, 90, 91
High prevalence of undiagnosed TB and delayed access to care in community	Randomized controlled trials (RCT) to investigate effective TB diagnostic algorithms, e.g. use of digital chest X‐ray screening	Implement active TB case finding in communities including community‐led initiatives	92, 93, 94
Few studies investigating risk of death associated with respiratory symptoms at community	Prospective studies investigating risk of death and risk factors and RCTs to modify risk of death Mortality surveys nested in longitudinal DHS sites	Improve death registration by setting‐up vital registration systems	95
Primary care			
High HIV prevalence among adults with cough	Operational research to achieve high coverage of accurate provider‐initiated testing and counselling	HIV testing for all health facility attendees Immediate ART initiation in accordance with guidelines and improve retention in care	5, 96
High TB prevalence among adults with cough	RCTs to investigate algorithms for TB screening, e.g. those that include Xpert MTB/RIF and digital chest radiography	TB screening for all health facility attendees for infection control and to improve care of patientsSystematic TB screening using of digital chest X‐ray screening and Xpert MTB/RIF	5
High risk of death among adults with cough	RCTs to investigate impact of new TB diagnostic tests to reduce risk of early death, potentially Xpert MTB/RIF Ultra	Operational recommendations are early HIV diagnosis, immediate ART and cotrimoxazole prophylaxis, and isoniazid preventive therapy if HIV positive	96, 97
Hospital			
Very high HIV prevalence among adults with cough	Operational research to achieve high coverage of accurate PITC	HIV testing for all health facility attendees Immediate ART initiation in accordance with guidelines and improve retention in care	5, 96
Very high TB prevalence among adults with cough	RCTs to investigate algorithms for TB screening in hospitals that include Xpert MTB/RIF and digital chest radiography	TB screening for all health facility attendees for infection control and to improve care of patients Isolation to reduce nosocomial spread	5
Very high risk of early death	RCTs to investigate impact of new TB diagnostic tests to reduce risk of early death, potentially Xpert MTB/RIF Ultra RCTs to investigate effect of antibiotics, nutrition and host‐directed therapies	Operational recommendations are early HIV diagnosis, immediate ART and cotrimoxazole prophylaxis, and isoniazid preventive therapy if HIV positive Use of urinary LAM to screen all HIV‐positive individuals for disseminated TB	60, 96, 97

TB, tuberculosis; DHS, demographic and health survey; ART, antiretroviral therapy; LAM, lipoarabinomannan; HIVST, HIV self‐testing.

This is the first systematic literature review to estimate the prevalence of HIV among adults with TB symptoms. However, other reviews have addressed related subjects. A previous systematic literature review assessed the diagnostic utility of symptoms for TB among people with HIV 3. This review reported that the best performing rule was the presence of any one of: current cough (any duration), fever, night sweats or weight loss with sensitivity of 78.9% (95% CI 58.3% to 90.9%) and specificity of 49.6% (95% CI 29.2% to 70.1%) 3. Another systematic literature review highlighted the importance of respiratory symptoms among HIV‐positive individuals 53, with a pooled odds ratio for the prevalence of cough of 3.05 (95% CI 2.24 to 4.16) among HIV‐positive compared to HIV‐negative individuals 53. HIV‐positive people remain at higher risk of respiratory symptoms even when started on ART, and are at increased risk of chronic lung disease from a variety of causes 54. Ideally, advice on smoking cessation and links to specialist services providing diagnosis and management of infectious and non‐infectious causes of lung disease should be included as part of routine HIV care, along with regular screening for TB 53.

As discussed above, our TB prevalence and mortality findings are not representative of low HIV prevalence settings. Within this limitation, however, we show the level of healthcare at which adults with TB symptoms present to be an important determinant of expected yield of TB on further screening. The median prevalence of undiagnosed TB was 6.9% for symptomatic adults identified at community level, 20.5% at primary care level and 34.8% for inpatients (Figure 2). At community as well as facility level, TB screening using symptoms and chest radiography combined with HIV testing for all with suspected TB, could then make an important contribution to early TB diagnosis as well as simultaneously providing HIV programmes with high yields of previously undiagnosed HIV. Oral kits packaged for HIV self‐testing provide safe, accurate and highly acceptable access to HIV diagnosis that can more easily be integrated into high‐throughput TB screening programmes than standard HIV testing services 55. Community‐based TB screening can lead to rapid reduction in undiagnosed infectious TB by reaching people who may otherwise remain undiagnosed for prolonged periods, potentially averting deaths and post‐tuberculous disability 56, 57.

The risk of early mortality among adults with TB symptoms also increased substantially with level of care at presentation, but with the major step‐up for this outcome at inpatient level. Death within two to six months of follow‐up was reported for 1.6% with TB symptoms at community level, 3.1% at primary care and 22.6% among hospital inpatients (Figure 2). All studies contributing to these estimates were from high HIV prevalence settings. The largest contributing study, conducted in outpatient facilities in South Africa, reported a threefold increase in the risk of death at six months for HIV‐positive compared to HIV‐patients are prompt HIV testing and linkage to cotrimoxazole and ART 58, 59 plus rapid diagnosis of TB disease, including use of urine lipoarabinomannan assay for HIV‐positive inpatients, followed by prompt TB treatment (Table 5) 60, 61. With mortality at such unacceptably high levels for such a common clinical presentation, intensified research and programmatic efforts aimed at reducing this risk should be of the highest priority, including intensified TB screening approaches, prophylactic broad‐spectrum antibiotics and host‐directed therapies (Table 5) 62, 63.

Limitations to this study include those that relate to selection of studies and those relating to approaches of analysis. There was the pronounced under‐representation of studies from low HIV as well as low TB prevalence settings. The included studies, mostly from high HIV prevalence settings, also potentially had different coverage of ART, and ART is likely to reduce prevalence of TB and also prevalence of TB symptoms. Due to our inclusion criteria, estimates of TB prevalence were limited to studies that also conducted HIV testing, thereby excluding most TB prevalence surveys. We only included studies published in English language; this may bias our estimates. There was considerable heterogeneity that was found in meta‐analyses for all three outcomes that could not be explained by differences in type of participants, geographical region or background burden of HIV and TB. Therefore, summary estimates from meta‐analyses were not presented. This may also be partly because statistical tests for heterogeneity do not work well with pooled proportions 64.

In conclusion, the findings from this systematic literature review illustrate the urgent need to improve the management of patients with TB symptoms in high HIV prevalence settings. HIV prevalence among adults with TB symptoms was high at all levels of healthcare, including in general population surveys, while TB prevalence and mortality risk increased substantially at primary care and hospital level respectively. The high yield of undiagnosed HIV should make both community‐ and facility‐based TB screening interventions of high interest to HIV programmes trying to reach UNAIDS 90‐90‐90 targets for HIV service coverage. TB programmes need to develop the necessary partnerships and expertise to ensure that HIV testing is provided alongside all TB screening interventions. More usable and effective high‐throughput TB screening algorithms are needed at all levels of the health system. The high risk of early death, most notable for inpatients, but also at primary care is an issue in urgent need of more research and intervention. Annual reporting to WHO of the coverage of HIV testing, yield of HIV, linkage to ART and treatment outcomes among people investigated for TB would increase the international visibility of this important patient group, encourage national programmes to implement existing international recommendations, and enable progress to be tracked over time.

## Competing interests

There are no conflicts of interest to declare.

## Authors’ contributions

MN, PM and ELC conceived and designed the experiments. MN, MM, JO, AGW, PM and ELC performed the experiments. MN, CF and KH analysed the data. MN, AGW, KH and ELC wrote the first draft of the manuscript. MN, MM, CF, AGW, JO, PM and ELC contributed to the writing of the manuscript. MN, AGW, KH, PM, JO, MM and ELC agreed with the manuscript's results and conclusions. MN, AGW, CF, PM and ELC assisted with design of analyses and interpretation of result

## Supporting information


**Table S1.** Search strategy
**Table S2.** Modified New‐castle Ottawa scale for non‐randomized studies
**Table S3.** Reasons for exclusion of studies with full‐text review (n = 230)
**Table S4.** Methodological quality assessment of included RCTs
**Table S5.** Methodological quality assessment of non‐randomized studies*
**Table S6.** Influence of study quality on HIV and TB estimates
**Figure S1.** Forest plot of HIV and TB prevalence in adults with symptoms of TB stratified by level of healthcare.
**Figure S2.** Forest plot of mortality risk in adults with symptoms of TB stratified by level of care

## References

[1] World Health Organization . Global Tuberculosis Report 2017. Switzerland: WHO; Geneva; 2017.

[2] Oxlade O , Menzies D . Putting numbers on the End TB Strategy—an impossible dream?. Lancet Glob Health. 2016;4(11):e764–5. 27720687 10.1016/S2214-109X(16)30268-6

[3] Getahun H , Kittikraisak W , Heilig CM , Corbett EL , Ayles H , Cain KP , *et al* Development of a standardized screening rule for tuberculosis in people living with HIV in resource‐constrained settings: individual participant data meta‐analysis of observational studies. PLoS Med. 2011;8(1):e1000391. 21267059 10.1371/journal.pmed.1000391 PMC3022524

[4] Stover J , Bollinger L , Izazola JA , Loures L , DeLay P , Ghys PD . What is required to end the AIDS epidemic as a public health threat by 2030? The cost and impact of the fast‐track approach. PLoS ONE. 2016;11(5):e0154893. 27159260 10.1371/journal.pone.0154893 PMC4861332

[5] World Health Organization . WHO policy on collaborative TB/HIV activities: guidelines for national programmes and other stakeholders. Switzerland: WHO; Geneva; 2012. 23586124

[6] Waitt CJ , Squire SB . A systematic review of risk factors for death in adults during and after tuberculosis treatment. Int J Tuberc Lung Dis. 2011;15(7):871–85. 21496360 10.5588/ijtld.10.0352

[7] Straetemans M , Glaziou P , Bierrenbach AL , Sismanidis C , van der Werf MJ . Assessing tuberculosis case fatality ratio: a meta‐analysis. PLoS ONE. 2011;6(6):e20755. 21738585 10.1371/journal.pone.0020755 PMC3124477

[8] Takarinda KC , Sandy C , Masuka N , Hazangwe P , Choto RC , Mutasa‐Apollo T , *et al* Factors associated with mortality among patients on TB treatment in the southern region of Zimbabwe, 2013. Tuberc Res Treat. 2017;2017:6232071. 28352474 10.1155/2017/6232071 PMC5352882

[9] Macpherson P , Dimairo M , Bandason T , Zezai A , Munyati SS , Butterworth AE , *et al* Risk factors for mortality in smear‐negative tuberculosis suspects: a cohort study in Harare, Zimbabwe. Int J Tuberc Lung Dis. 2011;15(10):1390–6. 22283900 10.5588/ijtld.11.0056 PMC3272461

[10] World Health Organization . Systematic screening for active tuberculosis: principles and recommendations. Geneva, Switzerland: WHO; 2013. Contract No.: 1 September 2015. 25996015

[11] World Health Organization . Global tuberculosis report 2016. Geneva, Switzerland: WHO; 2016.

[12] Shapiro AE , Chakravorty R , Akande T , Lönnroth K , Golub JE . A systematic review of the number needed to screen to detect a case of active tuberculosis in different risk groups. World Health Organization. 2013.

[13] Higgins JP , Green S . Cochrane handbook for systematic reviews of interventions. Hoboken, New Jersey: John Wiley & Sons; 2011.

[14] Kumar AM , Gupta D , Kumar A , Gupta RS , Kanchar A , Rao R , *et al* HIV testing among patients with presumptive tuberculosis: how do we implement in a routine programmatic setting? Results of a large operational research from India. PLoS ONE. 2016;11(5):e0156487. 27244055 10.1371/journal.pone.0156487 PMC4887014

[15] Ayles H , Schaap A , Nota A , Sismanidis C , Tembwe R , De Haas P , *et al* Prevalence of tuberculosis, HIV and respiratory symptoms in two Zambian communities: implications for tuberculosis control in the era of HIV. PLoS ONE. 2009;4(5):e5602. 19440346 10.1371/journal.pone.0005602 PMC2680044

[16] Corbett EL , Zezai A , Cheung YB , Bandason T , Dauya E , Munyati SS , *et al* Provider‐initiated symptom screening for tuberculosis in Zimbabwe: diagnostic value and the effect of HIV status. Bull World Health Organ. 2010;88(1):13–21. 20428349 10.2471/BLT.08.055467 PMC2802433

[17] Deribew A , Abebe G , Apers L , Abdissa A , Deribe F , Woldemichael K , *et al* Prevalence of pulmonary TB and spoligotype pattern of Mycobacterium tuberculosis among TB suspects in a rural community in Southwest Ethiopia. BMC Infect Dis. 2012;12:54. 22414165 10.1186/1471-2334-12-54 PMC3378444

[18] Lorent N , Choun K , Thai S , Kim T , Huy S , Pe R , *et al* Community‐based active tuberculosis case finding in poor urban settlements of Phnom Penh, Cambodia: a feasible and effective strategy. PLoS ONE. 2014;9(3):e92754. 24675985 10.1371/journal.pone.0092754 PMC3968028

[19] Kranzer K , Lawn SD , Meyer‐Rath G , Vassall A , Raditlhalo E , Govindasamy D , *et al* Feasibility, yield, and cost of active tuberculosis case finding linked to a mobile HIV service in Cape Town, South Africa: a cross‐sectional study. PLoS Med. 2012;9(8):e1001281. 22879816 10.1371/journal.pmed.1001281 PMC3413719

[20] Nliwasa M , MacPherson P , Mukaka M , Mdolo A , Mwapasa M , Kaswaswa K , *et al* High mortality and prevalence of HIV and tuberculosis in adults with chronic cough in Malawi: a cohort study. Int J Tuberc Lung Dis. 2016;20:202–10. 26792472 10.5588/ijtld.15.0388 PMC4711670

[21] Sekandi JN , List J , Luzze H , Yin XP , Dobbin K , Corso PS , *et al* Yield of undetected tuberculosis and human immunodeficiency virus coinfection from active case finding in urban Uganda. Int J Tuberc Lung Dis. 2014;18(1):13–9. 24365547 10.5588/ijtld.13.0129 PMC5454493

[22] Bjerregaard‐Andersen M , da Silva ZJ , Ravn P , Ruhwald M , Andersen PL , Sodemann M , *et al* Tuberculosis burden in an urban population: a cross sectional tuberculosis survey from Guinea Bissau. BMC Infect Dis. 2010;10(1):96. 20398388 10.1186/1471-2334-10-96 PMC2860354

[23] Boehme CC , Nicol MP , Nabeta P , Michael JS , Gotuzzo E , Tahirli R , *et al* Feasibility, diagnostic accuracy, and effectiveness of decentralised use of the Xpert MTB/RIF test for diagnosis of tuberculosis and multidrug resistance: a multicentre implementation study. Lancet. 2011;377(9776):1495–505. 21507477 10.1016/S0140-6736(11)60438-8 PMC3085933

[24] Churchyard GJ , Stevens WS , Mametja LD , McCarthy KM , Chihota V , Nicol MP , *et al* Xpert MTB/RIF versus sputum microscopy as the initial diagnostic test for tuberculosis: a cluster‐randomised trial embedded in South African roll‐out of Xpert MTB/RIF. Lancet Glob Health. 2015;3(8):e450–7. 26187490 10.1016/S2214-109X(15)00100-X

[25] Cox HS , Mbhele S , Mohess N , Whitelaw A , Muller O , Zemanay W , *et al* Impact of Xpert MTB/RIF for TB diagnosis in a primary care clinic with high TB and HIV prevalence in South Africa: a pragmatic randomised trial. PLoS Med. 2014;11(11):e1001760. 25423041 10.1371/journal.pmed.1001760 PMC4244039

[26] Hanrahan CF , Clouse K , Bassett J , Mutunga L , Selibas K , Stevens W , *et al* The patient impact of point‐of‐care vs. Laboratory placement of XpertW MTB/RIF. Int J Tuberc Lung Dis. 2015;19(7):811–6. 26056107 10.5588/ijtld.15.0013 PMC4869324

[27] Hanrahan CF , Selibas K , Deery CB , Dansey H , Clouse K , Bassett J , *et al* Time to treatment and patient outcomes among TB suspects screened by a single point‐of‐care xpert MTB/RIF at a primary care clinic in Johannesburg, South Africa. PLoS ONE. 2013;8(6):e65421. 23762367 10.1371/journal.pone.0065421 PMC3675091

[28] Muyoyeta M , Moyo M , Kasese N , Ndhlovu M , Milimo D , Mwanza W , *et al* Implementation research to inform the use of Xpert MTB/RIF in primary health care facilities in high TB and HIV settings in resource constrained settings. PLoS ONE. 2015;10(6):e0126376. 26030301 10.1371/journal.pone.0126376 PMC4451006

[29] Nliwasa M , MacPherson P , Chisala P , Kamdolozi M , Khundi M , Kaswaswa K , *et al* The sensitivity and specificity of loop‐mediated isothermal amplification (LAMP) assay for tuberculosis diagnosis in adults with chronic cough in Malawi. PLoS ONE. 2016;11(5):e0155101. 27171380 10.1371/journal.pone.0155101 PMC4865214

[30] Scott LE , McCarthy K , Gous N , Nduna M , Van Rie A , Sanne I , *et al* Comparison of Xpert MTB/RIF with other nucleic acid technologies for diagnosing pulmonary tuberculosis in a high HIV prevalence setting: a prospective study. PLoS Med. 2011;8(7):e1001061. 21814495 10.1371/journal.pmed.1001061 PMC3144192

[31] Naik B , Kumar A , Kanchar A , Rangaraju C , Deepak KG , Bhat P , *et al*, editors. HIV testing among presumptive tuberculosis cases in routine implementation, Karnataka, India. 44th World Conference on Lung Health of the International Union Against Tuberculosis and Lung Disease (The Union); 2013; Paris, France.

[32] Achanta S , Kumar AM , Nagaraja SB , Jaju J , Shamrao SR , Uppaluri R , *et al* Feasibility and effectiveness of provider initiated HIV testing and counseling of TB suspects in Vizianagaram district, South India. PLoS ONE. 2012;7(7):e41378. 22844467 10.1371/journal.pone.0041378 PMC3402476

[33] Naik B , Kumar Mv A , Lal K , Doddamani S , Krishnappa M , Inamdar V , *et al* HIV prevalence among persons suspected of tuberculosis: policy implications for India. J Acquir Immune Defic Syndr. 2012;59(4):e72–6. 22193775 10.1097/QAI.0b013e318245c9df

[34] Deribew A , Negussu N , Kassahun W , Apers L , Colebunders R . Uptake of provider‐initiated counselling and testing among tuberculosis suspects, Ethiopia. Int J Tuberc Lung Dis. 2010;14(11):1442–6. 20937185

[35] Odhiambo J , Kizito W , Njoroge A , Wambua N , Nganga L , Mburu M , *et al* Provider‐initiated HIV testing and counselling for TB patients and suspects in Nairobi, Kenya. Int J Tuberc Lung Dis. 2008;12 3 Suppl 1:63–8. 18302825

[36] Srikantiah P , Lin R , Walusimbi M , Okwera A , Luzze H , Whalen CC , *et al* Elevated HIV seroprevalence and risk behavior among Ugandan TB suspects: implications for HIV testing and prevention. Int J Tuberc Lung Dis. 2007;11(2):168–74. 17263287 PMC2846511

[37] Van Rie A , Clouse K , Hanrahan C , Selibas K , Sanne I , Williams S , *et al* High uptake of systematic HIV counseling and testing and TB symptom screening at a primary care clinic in South Africa. PLoS ONE. 2014;9(9):e105428. 25268851 10.1371/journal.pone.0105428 PMC4182031

[38] Bates M , Ahmed Y , Chilukutu L , Tembo J , Cheelo B , Sinyangwe S , *et al* Use of the Xpert^®^ MTB/RIF assay for diagnosing pulmonary tuberculosis comorbidity and multidrug‐resistant TB in obstetrics and gynaecology inpatient wards at the University Teaching Hospital, Lusaka, Zambia. Trop Med Int Health. 2013;18(9):1134–40. 23834035 10.1111/tmi.12145 PMC4016757

[39] O'Grady J , Bates M , Chilukutu L , Mzyece J , Cheelo B , Chilufya M , *et al* Evaluation of the Xpert MTB/RIF assay at a tertiary care referral hospital in a setting where tuberculosis and HIV infection are highly endemic. Clin Infect Dis. 2012;55(9):1171–8. 22806590 10.1093/cid/cis631

[40] Shah M , Variava E , Holmes CB , Coppin A , Golub JE , McCallum J , *et al* Diagnostic accuracy of a urine lipoarabinomannan test for tuberculosis in hospitalized patients in a High HIV prevalence setting. J Acquir Immune Defic Syndr. 2009;52(2):145–51. 19692904 10.1097/QAI.0b013e3181b98430 PMC2815254

[41] Yoon C , Cattamanchi A , Davis JL , Worodria W , den Boon S , Kalema N , *et al* Impact of Xpert MTB/RIF testing on tuberculosis management and outcomes in hospitalized patients in Uganda. PLoS ONE. 2012;7(11):e48599. 23139799 10.1371/journal.pone.0048599 PMC3490868

[42] Ministry of health and social welfare . The first national tuberculosis prevalence survey in the United Republic of Tanzania final report. 2013.

[43] Gawa LG , Reid T , Edginton ME , Van Lettow M , Joshua M , Harries AD . Diagnostic management and outcomes of pulmonary tuberculosis suspects admitted to a central hospital in Malawi. Public Health Action. 2011;1(1):2–5. 26392925 10.5588/pha.11.0007 PMC4547185

[44] van Lettow M , Bedell R , Maosa S , Phiri K , Chan AK , Mwinjiwa E , *et al* Outcomes and diagnostic processes in outpatients with presumptive tuberculosis in Zomba District, Malawi. PLoS ONE. 2015;10(11):e0141414. 26556045 10.1371/journal.pone.0141414 PMC4640882

[45] Mulder C , Mgode GF , Ellis H , Valverde E , Beyene N , Cox C , *et al* Accuracy of giant African pouched rats for diagnosing tuberculosis: comparison with culture and Xpert MTB/RIF. Trop Med Int Health. 2017;22 Supplement 1:357. 10.5588/ijtld.17.0139 29037292

[46] Hamilton C , Reddy E , Lancaster K , Stout JE , Njau B , Morpeth S , *et al* High mortality in patients with TB symptoms in Moshi, Tanzania: considerations for TB diagnostics, lung health and health systems strengthening. Am J Respir Crit Care Med. 2010;181:A3144.

[47] Morse M , Kessler J , Albrecht S , Kim R , Thakur R , Nthobatsang R , *et al* Induced sputum improves the diagnosis of pulmonary tuberculosis in hospitalized patients in Gaborone, Botswana. Int J Tuberc Lung Dis. 2008;12(11):1279–85. 18926038

[48] Nliwasa M , MacPherson P , Mukaka M , Mdolo A , Mwapasa M , Kaswaswa K , *et al* High mortality and prevalence of HIV and tuberculosis in adults with chronic cough in Malawi: a cohort study. Int J Tuberc Lung Dis. 2016;20(2):202–10. 26792472 10.5588/ijtld.15.0388 PMC4711670

[49] Rwanda Ministry of Health . Report of the first national pulmonary tuberculosis prevalence survey in Rwanda. 2014.

[50] Dlodlo RA , Hwalima ZE , Sithole S , Takarinda KC , Tayler‐Smith K , Harries AD . Are HIV‐positive presumptive tuberculosis patients without tuberculosis getting the care they need in Zimbabwe? Public Health Action. 2015;5(4):217–21. 26767174 10.5588/pha.15.0036 PMC4682612

[51] Mwansa‐Kambafwile J , Maitshotlo B , Black A . Microbiologically confirmed tuberculosis: factors associated with pre‐treatment loss to follow‐up, and time to treatment initiation. PLoS ONE. 2017;12(1):e0168659. 28068347 10.1371/journal.pone.0168659 PMC5222612

[52] Malawi National AIDS Commission . National Strategic Plan for HIV and AIDS (2015–2020). 2014.

[53] Brown J , Roy A , Harris R , Filson S , Johnson M , Abubakar I , *et al* Respiratory symptoms in people living with HIV and the effect of antiretroviral therapy: a systematic review and meta‐analysis. Thorax. 2017;72(4):355–66. 27965402 10.1136/thoraxjnl-2016-208657 PMC5520276

[54] Rylance J , McHugh G , Metcalfe J , Mujuru H , Nathoo K , Wilmore S , *et al* Chronic lung disease in HIV‐infected children established on antiretroviral therapy. AIDS. 2016;30(18):2795–803. 27662546 10.1097/QAD.0000000000001249 PMC5106089

[55] MacPherson P , Lalloo DG , Webb EL , Maheswaran H , Choko AT , Makombe SD , *et al* Effect of optional home initiation of HIV care following HIV self‐testing on antiretroviral therapy initiation among adults in Malawi: a randomized clinical trial. JAMA. 2014;312(4):372–9. 25038356 10.1001/jama.2014.6493 PMC4118051

[56] Corbett EL , Bandason T , Cheung YB , Makamure B , Dauya E , Munyati SS , *et al* Prevalent infectious tuberculosis in Harare, Zimbabwe: burden, risk factors and implications for control. Int J Tuberc Lung Dis. 2009;13(10):1231–7. 19793427 PMC3374846

[57] Wood R , Middelkoop K , Myer L , Grant AD , Whitelaw A , Lawn SD , *et al* Undiagnosed tuberculosis in a community with high HIV prevalence: implications for tuberculosis control. Am J Respir Crit Care Med. 2007;175(1):87–93. 16973982 10.1164/rccm.200606-759OC PMC1899262

[58] Abdool Karim SS , Naidoo K , Grobler A , Padayatchi N , Baxter C , Gray A , *et al* Timing of initiation of antiretroviral drugs during tuberculosis therapy. N Engl J Med. 2010;362(8):697–706. 20181971 10.1056/NEJMoa0905848 PMC3076221

[59] Blanc FX , Sok T , Laureillard D , Borand L , Rekacewicz C , Nerrienet E , *et al* Earlier versus later start of antiretroviral therapy in HIV‐infected adults with tuberculosis. N Engl J Med. 2011;365(16):1471–81. 22010913 10.1056/NEJMoa1013911 PMC4879711

[60] Peter JG , Zijenah LS , Chanda D , Clowes P , Lesosky M , Gina P , *et al* Effect on mortality of point‐of‐care, urine‐based lipoarabinomannan testing to guide tuberculosis treatment initiation in HIV‐positive hospital inpatients: a pragmatic, parallel‐group, multicountry, open‐label, randomised controlled trial. Lancet. 2016;387(10024):1187–97. 26970721 10.1016/S0140-6736(15)01092-2

[61] Gupta‐Wright A , Corbett EL , vanOosterhout JJ , Wilson DK , Grint D , Alufandika‐Moyo M , *et al* Urine‐based screening for tuberculosis: a randomised trial in HIV‐positive inpatients. Conference on Retroviruses and Opportunistic Infections 4–7 March; Boston, Massachusetts 2018.

[62] Hakim J , Musiime V , Szubert AJ , Siika A , Mallewa J , Agutu C , *et al* Enhanced infection prophylaxis reduces mortality in severely immunosuppressed HIV‐infected adults and older children initiating antiretroviral therapy in Kenya, Malawi, Uganda, and Zimbabwe: the REALITY trial [abstract FRAB0101LB]. 21st International AIDS Conference Durban, South Africa; 2016.

[63] Zumla A , Rao M , Wallis RS , Kaufmann SH , Rustomjee R , Mwaba P , *et al* Host‐directed therapies for infectious diseases: current status, recent progress, and future prospects. Lancet Infect Dis. 2016;16(4):e47–63. 27036359 10.1016/S1473-3099(16)00078-5 PMC7164794

[64] Mills EJ , Jansen JP , Kanters S . Heterogeneity in meta‐analysis of FDG‐PET studies to diagnose lung cancer. JAMA. 2015;313(4):419. 10.1001/jama.2014.16482 25626041

[65] Rivera VR , Jean‐Juste MA , Gluck SC , Reeder HT , Sainristil J , Julma P , *et al* Diagnostic yield of active case finding for tuberculosis and HIV at the household level in slums in Haiti. Int J Tuberc Lung Dis. 2017;21(11):1140–6. 29037294 10.5588/ijtld.17.0049 PMC5902800

[66] Uganda Ministry of Health . The Uganda National Tuberculosis Prevalence Survey, 2014–2015 ‐ Survey Report. 2017.

[67] Yotebieng M , Wenzi LK , Basaki E , Batumbula ML , Tabala M , Mungoyo E , *et al*, editors. PITC of tuberculosis suspects and prevalence of HIV among tuberculosis suspects in Kinshasa and Kisangani, Democratic Republic of Congo. 44rd World Conference OM Lung Health of the International Union Against Tuberculosis and Lung Disease (The Union); 2013; Paris, France.

[68] Sahle SN , Asress DT , Tullu KD , Weldemariam AG , Tola HH , Awas YA , *et al* Performance of point‐of‐care urine test in diagnosing tuberculosis suspects with and without HIV infection in selected peripheral health settings of Addis Ababa, Ethiopia. BMC Res Notes. 2017;10(1):74. 28137314 10.1186/s13104-017-2404-4 PMC5282652

[69] Rudolf F , Wagner AJ , Back FM , Gomes VF , Aaby P , Ostergaard L , *et al* Tuberculosis case finding and mortality prediction: added value of the clinical TBscore and biomarker suPAR. Int J Tuberc Lung Dis. 2017;21(1):67–72. 28157467 10.5588/ijtld.16.0404

[70] Kivihya‐Ndugga LE , van Cleeff MR , Githui WA , Nganga LW , Kibuga DK , Odhiambo JA , *et al* A comprehensive comparison of Ziehl‐Neelsen and fluorescence microscopy for the diagnosis of tuberculosis in a resource‐poor urban setting. Int J Tuberc Lung Dis. 2003;7(12):1163–71. 14677891

[71] Munthali L , Mwaungulu JN , Munthali K , Bowie C , Crampin AC . Using tuberculosis suspects to identify patients eligible for antiretroviral treatment. Int J Tuberc Lung Dis. 2006;10(2):199–202. 16499261

[72] Brunet L , Pai M , Davids V , Ling D , Paradis G , Lenders L , *et al* High prevalence of smoking among patients with suspected tuberculosis in South Africa. Eur Respir J. 2011;38(1):139–46. 21148230 10.1183/09031936.00137710 PMC5454478

[73] Theron G , Peter J , van Zyl‐Smit R , Mishra H , Streicher E , Murray S , *et al* Evaluation of the Xpert MTB/RIF assay for the diagnosis of pulmonary tuberculosis in a high HIV prevalence setting. Am J Respir Crit Care Med. 2011;184(1):132–40. 21493734 10.1164/rccm.201101-0056OC

[74] Geldenhuys HD , Whitelaw A , Tameris MD , Dv As , Luabeya KKA , Mahomed H , *et al* A controlled trial of sputum induction and routine collection methods for TB diagnosis in a South African community. Eur J Clin Microbiol Infect Dis. 2014;33(12):2259–66. 25022447 10.1007/s10096-014-2198-4 PMC4229508

[75] Munyati SS , Dhoba T , Makanza ED , Mungofa S , Wellington M , Mutsvangwa J , *et al* Chronic cough in primary health care attendees, Harare, Zimbabwe: diagnosis and impact of HIV infection. Clin Infect Dis. 2005;40(12):1818–27. 15909272 10.1086/429912

[76] Legesse M , Ameni G , Mamo G , Medhin G , Bjune G , Abebe F . Performance of QuantiFERON‐TB Gold In‐Tube (QFTGIT) for the diagnosis of Mycobacterium tuberculosis (Mtb) infection in Afar Pastoralists, Ethiopia. BMC Infect Dis. 2010;10:354. 21162756 10.1186/1471-2334-10-354 PMC3009640

[77] Belay M , Bjune G , Abebe F . Prevalence of tuberculosis, HIV, and TB‐HIV co‐infection among pulmonary tuberculosis suspects in a predominantly pastoralist area, northeast Ethiopia. Glob Health Action. 2015;8:27949. 26689454 10.3402/gha.v8.27949 PMC4685972

[78] Adjei AA , Adiku TK , Ayeh‐Kumi PF , Hesse IF . Prevalence of human immunodeficiency virus infection among tuberculosis suspect patients in Accra, Ghana. West Afr J Med. 2006;25(1):38–41. 16722357 10.4314/wajm.v25i1.28243

[79] Kaur P , Sharma P , Aggarwal A . HIV positivity in TB suspects–an observational, non‐randomized study. Indian J Tuberc. 2013;60(1):59–60. 23540091

[80] Aliyu G , El‐Kamary SS , Abimiku A , Brown C , Tracy K , Hungerford L , *et al* Prevalence of non‐tuberculous mycobacterial infections among tuberculosis suspects in Nigeria. PLoS ONE. 2013;8(5):e63170. 23671669 10.1371/journal.pone.0063170 PMC3650061

[81] Okonkwo RC , Anyabolu AE , Ifeanyichukwu M , Kalu SO . Prevalence of HIV infection in pulmonary tuberculosis suspects; assessing the Nnamdi Azikiwe University Teaching Hospital, Nnewi, Nigeria. Adv Life Sci Technol. 2013;14:1.

[82] Rachow A , Zumla A , Heinrich N , Rojas‐Ponce G , Mtafya B , Reither K , *et al* Rapid and accurate detection of Mycobacterium tuberculosis in sputum samples by Cepheid Xpert MTB/RIF assay–a clinical validation study. PLoS ONE. 2011;6(6):e20458. 21738575 10.1371/journal.pone.0020458 PMC3126807

[83] Kawkitinarong K , Suwanpimolkul G , Kateruttanakul P , Manosuthi W , Ubolyam S , Sophonphan J , *et al* Real‐life clinical practice of using the Xpert MTB/RIF assay in Thailand. Clin Infect Dis. 2017;2:S171–8. 10.1093/cid/cix151 28475796

[84] Nanta S , Kantipong P , Pathipvanich P , Ruengorn C , Tawichasri C , Patumanond J . Diagnostic value of two rapid immunochromatographic tests for suspected tuberculosis diagnosis in clinical practice. J Med Assoc Thai. 2011;94(10):1198–204. 22145504

[85] Pinyopornpanish K , Chaiwarith R , Pantip C , Keawvichit R , Wongworapat K , Khamnoi P , *et al* Comparison of Xpert MTB/RIF assay and the conventional sputum microscopy in detecting Mycobacterium tuberculosis in Northern Thailand. Tuberc Res Treat. 2015;2015:571782. 26064681 10.1155/2015/571782 PMC4430669

[86] Talbot EA , Hay Burgess DC , Hone NM , Iademarco MF , Mwasekaga MJ , Moffat HJ , *et al* Tuberculosis serodiagnosis in a predominantly HIV‐infected population of hospitalized patients with cough, Botswana, 2002. Clin Infect Dis. 2004;39(1):e1–7. 15206074 10.1086/421388

[87] Hirao S , Yassin MA , Khamofu HG , Lawson L , Cambanis A , Ramsay A , *et al* Same‐day smears in the diagnosis of tuberculosis. Trop Med Int Health. 2007;12(12):1459–63. 18076552 10.1111/j.1365-3156.2007.01952.x

[88] Shah S , Demissie M , Lambert L , Ahmed J , Leulseged S , Kebede T , *et al* Intensified tuberculosis case finding among HIV‐Infected persons from a voluntary counseling and testing center in Addis Ababa, Ethiopia. JAIDS. 2009;50(5):537–45. 19223783 10.1097/QAI.0b013e318196761c

[89] Jones‐Lopez E , Manabe YC , Palaci M , Kayiza C , Armstrong D , Nakiyingi L , *et al* Prospective cross‐sectional evaluation of the small membrane filtration method for diagnosis of pulmonary tuberculosis. J Clin Microbiol. 2014;52(7):2513–20. 24808236 10.1128/JCM.00642-14 PMC4097702

[90] Choko AT , MacPherson P , Webb EL , Willey BA , Feasy H , Sambakunsi R , *et al* Uptake, accuracy, safety, and linkage into care over two years of promoting annual self‐testing for HIV in Blantyre, Malawi: a community‐based prospective study. PLoS Med. 2015;12(9):e1001873. 26348035 10.1371/journal.pmed.1001873 PMC4562710

[91] Labhardt ND , Motlomelo M , Cerutti B , Pfeiffer K , Kamele M , Hobbins MA , *et al* Home‐based versus mobile clinic HIV testing and counseling in rural Lesotho: a cluster‐randomized trial. PLoS Med. 2014;11(12):e1001768. 25513807 10.1371/journal.pmed.1001768 PMC4267810

[92] Kranzer K , Afnan‐Holmes H , Tomlin K , Golub JE , Shapiro AE , Schaap A , *et al* The benefits to communities and individuals of screening for active tuberculosis disease: a systematic review. Int J Tuberc Lung Dis. 2013;17(4):432–46. 23485377 10.5588/ijtld.12.0743

[93] Corbett EL , Bandason T , Duong T , Dauya E , Makamure B , Churchyard GJ , *et al* Comparison of two active case‐finding strategies for community‐based diagnosis of symptomatic smear‐positive tuberculosis and control of infectious tuberculosis in Harare, Zimbabwe (DETECTB): a cluster‐randomised trial. Lancet. 2010;376(9748):1244–53. 20923715 10.1016/S0140-6736(10)61425-0 PMC2956882

[94] Ayles H , Muyoyeta M , Du Toit E , Schaap A , Floyd S , Simwinga M , *et al* Effect of household and community interventions on the burden of tuberculosis in southern Africa: the ZAMSTAR community‐randomised trial. Lancet. 2013;382(9899):1183–94. 23915882 10.1016/S0140-6736(13)61131-9

[95] Korenromp EL , Bierrenbach AL , Williams BG , Dye C . The measurement and estimation of tuberculosis mortality. Int J Tuberc Lung Dis. 2009;13(3):283–303. 19275787

[96] Lundgren JD , Babiker AG , Gordin F , Emery S , Grund B , Sharma S , *et al* Initiation of antiretroviral therapy in early asymptomatic HIV infection. N Engl J Med. 2015;373(9):795–807. 26192873 10.1056/NEJMoa1506816 PMC4569751

[97] Danel C , Moh R , Gabillard D , Badje A , Le Carrou J , Ouassa T , *et al* A trial of early antiretrovirals and isoniazid preventive therapy in Africa. N Engl J Med. 2015;373(9):808–22. 26193126 10.1056/NEJMoa1507198

