# Supplementary material for: High HIV and active tuberculosis prevalence and increased mortality risk in adults with symptoms of TB: a systematic review and meta‐analyses
Source: J Int AIDS Soc. 2018 Jul 31;21(7):e25162. doi: 10.1002/jia2.25162 (PMC6067081; doi:10.1002/jia2.25162)
Supplement: Supplementary file 1 — Table S1. Search strategy Table S2. Modified New‐castle Ottawa scale for non‐randomized studies Table S3. Reasons for exclusion of studies with full‐text review (n = 230) Table S4. Methodological quality assessment of included RCTs Table S5. Methodological quality assessment of non‐randomized studies* Table S6. Influence of study quality on HIV and TB estimates Figure S1. Forest plot of HIV and TB prevalence in adults with symptoms of TB stratified by level of healthcare. Figure S2. Forest plot of mortality risk in adults with symptoms of TB stratified by level of care [file JIA2-21-e25162-s001.docx]

**Supplementary material**

| **Concepts** | **Set** | **Search terms** |
| --- | --- | --- |
| Tuberculosis | 1-3 | exp *Tuberculosis/ OR Tuberculosis.mp. OR TB.mp |
|  | 4 | 1 or 2 or 3 |
| Cough | 5-9 | suspect*.mp OR presumpt*.mp OR probabl*.mp OR exp Cough/ OR cough.mp |
|  | 10 | 5 or 6 or 7 or 8 or 9 |
| HIV | 11-21 | exp HIV/ or exp HIV-2/ or exp HIV-1/ or exp HIV Infections/ OR human immunodeficiency virus.mp OR human immune deficiency virus.mp OR human immuno-deficiency virus.mp OR human immune-deficiency virus.mp OR acquired immunodeficiency syndrome.mp OR acquired immune deficiency syndrome.mp OR acquired immune-deficiency syndrome.mp OR HIV.mp. OR (HIV adj2 infect*).mp OR AIDS mp |
|  | 22 | 11 or 12 or 13 or 14 or 15 or 16 or 17 or 18 or 19 or 20 or 21 |
| Context of study | 23-55 | exp Mass Screening/ OR screen*.mp. OR exp Diagnosis/ OR diagnos*.mp. OR detect*.mp. OR (active adj1 case adj1 find*).mp. OR (enhanced adj1 case adj1 find*).mp. OR (new adj2 diagnos*).mp. OR (diagnos* adj2 evaluat*).mp. OR (diagnos* adj2 implement*).mp. OR (prevalence adj1 survey).mp. OR contact tracing.mp. OR microscop*.mp. OR (LED adj1 microscopy).mp. OR (smear adj1 microscopy).mp. OR "Xpert MTB/Rif".mp. OR exp Polymerase Chain Reaction/ or exp Molecular Diagnostic Techniques/ or exp Nucleic Acid Amplification Techniques/ OR Xpert.mp. OR x-ray.mp. OR exp Radiology/ or exp Radiography/ OR radiolog*.mp. Lipoarabinomannan.mp. OR Loop mediated isothermal amplif*.mp. OR exp Culture Techniques/ OR culture.mp OR MGIT.mp. OR randomized controlled trial.pt OR controlled clinical trial.pt OR randomized controlled trials/ OR random allocation/ OR double blind method/ OR Cohort Studies/ or Cohort Effect/ or cohort.mp OR Prospective Studies/ or Treatment Outcome/ |
|  | 56 | 23 or 24 or 25 or 26 or 27 or 28 or 29 or 30 or 31 or 32 or 33 or 34 or 35 or 36 or 37 or 38 or 39 or 40 or 41 or 42 or 43 or 44 or 45 or 46 or 47 or 48 or 49 or 50 or 51 or 52 or 53 or 54 or 55 |
|  | 57 | 4 or 10 |
|  | 58 | 22 and 57 |
|  | 59 | 56 and 58 |
|  | 60 | limit 59 to year="2003 -Current" |
|  | 61 | limit 78 to English language |

1. **Search strategy**

| **Domain** | **Item assessed** | **Risk of bias** |
| --- | --- | --- |
| **Selection** |  |  |
| Representativeness of cohort | 1. Participants are representative of the general population |  |
|  | 1. Study participants are representative sample of population | Low |
|  | 1. Participants are select group of population | High |
|  | 1. No description | Unclear |
| Selection of the cohort | 2. Consecutive participants were invited to participate |  |
|  | 1. Consecutive participants invited | Low |
|  | 1. Retrospective recruitment or assessment of clinic records | High |
|  | 1. No description | Unclear |
| Ascertainment of exposure | Not assessed |  |
| Outcome was absent at study start | 3. Demonstrate that outcome was not present at start (mortality) |  |
|  | 1. Yes, no history of other diseases that cause early death | Low |
|  | 1. No | High |
|  | Note: HIV and TB prevalence outcomes did not require prior absence |  |
| **Comparability** |  |  |
| Comparability of cohorts | Not assessed |  |
| **Outcome 1** |  |  |
| HIV testing method | 4. Method of ascertaining HIV status |  |
|  | 1. Biomedical test used | Low |
|  | 1. Link to medical records or verbal report | High |
|  | 1. No description | Unclear |
| HIV test uptake | 5. Uptake of HIV testing |  |
|  | 1. High uptake (>80%) | Low |
|  | 1. Low (<80%) | High |
|  | 1. No description (<50%) | Unclear |
| **Outcome 2** |  |  |
| Group for TB diagnosis | 6. Method for initial screening |  |
|  | 1. Objective method e.g. symptom screen questionnaire | Low |
|  | 1. Subjective method e.g. clinician judgement or referral | High |
|  | 1. Other or no description | Unclear |
| TB testing method | 7. Diagnosis of TB |  |
|  | 1. Bacteriological confirmation (culture/microscopy/Xpert MTB/RIF) | Low |
|  | 1. Clinical diagnosis | High |
|  | 1. No description | Unclear |
| TB testing uptake | 8. Participation rate of TB testing |  |
|  | 1. High participation (>80%) | Low |
|  | 1. Low participation (<80%) | High |
|  | 1. No description | Unclear |
| **Outcome 3** |  |  |
| Mortality assessment | 9. Assessment of mortality |  |
|  | 1. Assessment of death using study or hospital records* | Low |
|  | 1. Link to verbal autopsy reports or vital registration systems | High |
|  | 1. No description | Unclear |
| Follow-up completeness | 10. Adequacy of follow-up |  |
|  | 1. High participation (>80%) * | Low |
|  | 1. Low participation (<80%) | High |
|  | 1. No description | Unclear |

1. **Modified New-castle Ottawa scale for non-randomised studies**
2. **Reasons for exclusion of studies with full-text review (n=230)**

| **Reasons** | **Number of studies** | **References** |
| --- | --- | --- |
| **Reported on select participant groups** | **158 (68.7%)** |  |
| HIV positive individuals only | 102 | ^1-102^ |
| Patients with smear negative TB only | 17 | ^103-119^ |
| Household contacts of TB patients only | 10 | ^120-129^ |
| Other select groups ^a^ | 29 | ^130-158^ |
| **Disaggregated data not available** | **48 (20.9%)** |  |
| No data for adults with TB symptoms | 38 | ^159-196^ |
| HIV testing data for those with TB disease only | 10 | ^197-206^ |
| **Other reasons** | **24 (10.4%)** |  |
| Ineligible article types ^b^ | 7 | ^207-213^ |
| Duplicate studies | 5 | ^214-218^ |
| Insufficient data by conference abstracts | 8 | ^219-226^ |
| Reports on TB diagnosis on special samples ^c^ | 4 | ^227-230^ |
| **Total number of studies excluded** | **230 (100%)** |  |

^a^ Includes people who inject drugs, pregnant women, prisoners, gold miners and HIV negative patients only

^b^ Opinions, commentaries, reviews, qualitative design, basic science research, pure economic analysis studies

^c^ For example, broncho-alveolar lavage samples, and blood samples

1. **Methodological quality assessment of included RCTs**

| **Churchyard, 2015** |  |  |
| --- | --- | --- |
| **Domain** | **Judgement** | **Support for judgement** |
| Random sequence generation  (selection bias) | Low risk | Quote: “Laboratories were grouped into four strata, based on province, and randomised by a statistician using Stata to either the Xpert or the microscopy study group”  Comment: This cluster-randomised trial there was random allocation of clusters |
| Allocation concealment  (selection bias) | Low risk | This was a cluster-randomised trial, randomisation- the random sequence was not applicable for the recruitment of individual participants |
| Blinding of participants and personnel  (performance bias) | Unclear risk | Quote: “masking of participants’ group allocation was not possible because of the pragmatic trial design”  Comment: The effect of lack of blinding of participants in this cluster-randomised trial on the outcomes is unclear |
| Blinding of outcome assessment – HIV  (detection bias) | Low risk | No information was provided on whether people providing HIV testing were blinded, but probably randomisation of a TB test in this CRT does not affect HIV testing patterns |
| Blinding of outcome assessment – tuberculosis  (detection bias) | Low risk | Blinding of the TB testing use was not possible and relevant for this CRT design |
| Blinding of outcome assessment – All-cause mortality  (detection bias) | Low risk | Quote: “Study staff, who were unaware of the index test result, telephoned participants 1 week and 1, 2, and 4 months after enrolment to maintain contact and update locator information”  Comment: study staff ascertaining all-cause mortality were blinded |
| Incomplete outcome data – HIV  (Attrition bias) | High risk | 633/2324 (27.2%) in the Xpert arm and 481/2332 (20.6%) in the microscopy arm had unknown HIV status |
| Incomplete outcome data – tuberculosis  (Attrition bias) | Low risk | 2176/2324 (93.6%) in the Xpert arm and 2235/2332 (95.8%) in the microscopy arm were successfully tested for TB |
| Incomplete outcome data – all cause mortality  (Attrition bias) | Low risk | 2324/2344 (99.0%) in the Xpert arm and 2235/2332 (98.5%) in the microscopy arm had outcome of vital status |
| Selective reporting | Low risk | All outcomes set in protocol are presented |

**Table S4** Continued

| **Cox, 2014** |  |  |
| --- | --- | --- |
| **Domain** | **Judgement** | **Support for judgement** |
| Random sequence generation  (selection bias) | Low risk | Quote: “Randomisation occurred on a weekly  basis in order to allow efficient laboratory functioning and use of resources. Each week was randomly allocated as either Xpert or  routine diagnostic testing, with the schedule generated prior to the study (using Random.org) by the principal investigator.”  Comment: random sequence was generated well |
| Allocation concealment  (selection bias) | High risk | Quote: “Each week was randomly allocated as either Xpert or routine diagnostic testing, with the schedule generated prior to the study (using Random.org) by the principal investigator.”  Comment: the principal investigator had access to the random sequence this may introduce bias |
| Blinding of participants and personnel  (performance bias) | High risk | Quote: “Clinic staff and investigators were unblinded to the intervention.”  Comment: Probably due to the pragmatic nature of the trial it was not seen as necessary to do |
| Blinding of outcome assessment – HIV  (detection bias) | High risk | Probably due to the pragmatic nature of the trial it was not done (see quote above). |
| Blinding of outcome assessment – tuberculosis  (detection bias) | High risk | Probably due to the pragmatic nature of the trial it was not done (see quote above). |
| Blinding of outcome assessment – All-cause mortality  (detection bias) | High risk | Probably due to the pragmatic nature of the trial it was not done (see quote above). |
| Incomplete outcome data – HIV  (Attrition bias) | Low risk | 815/982 (83.0%) in the Xpert arm and 810/1003 (80.8%) in the routine arm had known HIV status |
| Incomplete outcome data – tuberculosis  (Attrition bias) | Low risk | 964/982 (98.2%) in the Xpert arm and 981/1003 (97.8%) in the routine arm had sputum sample available |
| Incomplete outcome data – all cause mortality  (Attrition bias) | Unclear risk | Quote: “Within 6 mo from presentation, 3.4% (33/982) of participants died in the Xpert arm and 3.8% (38/1,003) in the routine arm”  Comment: outcome of mortality assessed in intention to treat analysis but the numbers of those actually followed up is not reported |
| Selective reporting | Unclear risk | Quote: “Two of these secondary outcomes,  time to diagnosis and the number of clinic visits prior to treatment, are not reported on here, as data were of poor quality and inconsistently available.”  Comment: It is unclear how the same reason above affected reporting of other outcomes |

1. **Methodological quality assessment of non-randomised studies***

|  | **Outcome 1 - HIV testing** |  |  | **Outcome 2 -TB testing** |  | **Outcome 3 -Mortality** |  | **Overall risk of bias for outcomes** |  |  |
| --- | --- | --- | --- | --- | --- | --- | --- | --- | --- | --- |
| **Author and year, setting** | **Method of HIV testing** | **Uptake of HIV testing** | **Use of symptom screening questionnaire** | **Methods of diagnosis of TB** | **Participation rate of TB testing** | **Assessment of mortality** | **Adequacy of follow-up** | **HIV prevalence** | **TB prevalence** | **Mortality risk** |
| Ayles, 2009, community | Low risk | Low risk | Low risk | Low risk | Low risk | NA | NA | Low risk | Low risk | NA |
| B-Andersen, 2009, community | High risk | High risk | Low risk | Low risk | Low risk | NA | NA | High risk | Low risk | NA |
| Corbett, 2010, community | Low risk | Low risk | Low risk | Low risk | Low risk | NA | NA | Low risk | Low risk | NA |
| Deribew, 2012, community | Low risk | Low risk | Low risk | Low risk | Low risk | NA | NA | Low risk | Low risk | NA |
| Kranzer, 2012, community | Low risk | Low risk | Low risk | Low risk | Low risk | NA | NA | Low risk | Low risk | NA |
| Lorent, 2012, community | Unclear risk | Unclear risk | Low risk | Low risk | Low risk | NA | NA | Unclear risk | Low risk | NA |
| Nliwasa, 2016, community | Low risk | Low risk | High risk | Low risk | Low risk | High risk | High risk | Low risk | Low risk | High risk |
| Rwanda, 2012, community | Low risk | Low risk | Low risk | Low risk | Low risk | NA | NA | Low risk | Low risk | NA |
| Tanzania, 2012, community | Low risk | High risk | Low risk | Low risk | Low risk | NA | NA | Unclear risk | Low risk | NA |
| Sekandi, 2014, community | Low risk | Low risk | Low risk | Low risk | Low risk | NA | NA | Low risk | Low risk | NA |
| Uganda, 2017, community | Low risk | Low risk | Low risk | Low risk | Low risk | NA | NA | Low risk | Low risk | NA |
| Rivera, Haiti, 2017 | Low risk | Low risk | Low risk | Low risk | Low risk | NA | NA | Low risk | Low risk | NA |
| Kumar, 2017, primary care | Low risk | Unclear risk | High risk | Unclear risk | NA | NA | NA | Low risk | NA | NA |
| Achanta, 2012, primary care | High risk | Low risk | High risk | Low risk | Low risk | NA | NA | High risk | Low risk | NA |
| Boehme, India, 2011, primary care | Unclear risk | Unclear risk | High risk | Low risk | Low risk | NA | NA | Unclear risk | Low risk | NA |
| Brunet, 2011, primary care | Low risk | Low risk | High risk | Low risk | Low risk | NA | NA | Low risk | Low risk | NA |
| Deribew, 2010, primary care | Low risk | High risk | High risk | Unclear risk | Low risk | NA | NA | High risk | Unclear risk | NA |
| Geldenhuys, 2014, primary care | Low risk | Low risk | High risk | Low risk | Low risk | NA | NA | Low risk | Low risk | NA |
| Hanrahan, 2013, primary care | Low risk | Low risk | Low risk | Low risk | Low risk | High risk | High risk | Low risk | Low risk | High risk |
| Hanrahan, 2015, primary care | Low risk | Low risk | Low risk | Low risk | Low risk | NA | NA | Low risk | Low risk | NA |
| K-Ndugga, 2003, primary care | Low risk | Unclear risk | High risk | Low risk | Low risk | NA | NA | Low risk | Low risk | NA |

*Only key domains are presented here a full excel table is available at the following link: <https://figshare.com/s/ead4d9c069268564d547>

(Continued)

**Table S5** (Continued)

|  | **Outcome 1 - HIV testing** |  |  | **Outcome 2 -TB testing** |  | **Mortality Assessment** |  | **Overall risk of bias for outcomes** |  |  |
| --- | --- | --- | --- | --- | --- | --- | --- | --- | --- | --- |
| **Author and year, setting** | **Method of HIV testing** | **Uptake of HIV testing** | **Use of symptom screening questionnaire** | **Methods of diagnosis of TB** | **Participation rate of TB testing** | **Assessment of mortality** | **Adequacy of follow-up** | **HIV prevalence** | **TB prevalence** | **Mortality risk** |
| Naik, 2012, primary care | Low risk | Low risk | High risk | Low risk | Low risk | NA | NA | Low risk | Low risk | NA |
| Nliwasa, 2016, primary care | Low risk | Low risk | Low risk | Low risk | Low risk | Low risk | Low risk | Low risk | Low risk | Low risk |
| Odhiambo, 2008, primary care | Low risk | Low risk | High risk | Low risk | Low risk | NA | NA | Low risk | Low risk | NA |
| Srikantiah, 2007, primary care | Low risk | Low risk | High risk | Low risk | Low risk | NA | NA | Low risk | Low risk | NA |
| Theron, 2011, primary care | Low risk | Low risk | High risk | Low risk | Low risk | NA | NA | Low risk | Low risk | NA |
| Van Rie, 2014, primary care | Low risk | Low risk | Low risk | Low risk | Low risk | NA | NA | Low risk | Low risk | NA |
| Scott, 2011, primary care | Low risk | Low risk | High risk | Low risk | Low risk | Low risk | High risk | Low risk | Low risk | High risk |
| Yotebieng, 2013, Primary care | Low risk | Low risk | High risk | Unclear risk | Low risk | NA | NA | Low risk | Unclear risk | NA |
| Rudolf, 2017, primary care | Low risk | Low risk | High risk | Unclear risk | Low risk | NA | NA | Low risk | Unclear risk | Low risk |
| Sahle, 2017, primary care | Low risk | Low risk | High risk | Low risk | Low risk | NA | NA | Low risk | Low risk | NA |
| Muyoyeta, 2015, primary care | Low risk | Low risk | High risk | Low risk | Low risk | NA | NA | Low risk | Low risk | NA |
| Munthali, 2006, primary care | Low risk | Low risk | High risk | Unclear risk | Low risk | NA | NA | Low risk | Unclear risk | NA |
| Dlodlo, 2015, primary care | Low risk | Low risk | NA | NA | NA | NA | NA | Low risk | NA | NA |
| Munyati, 2009, primary care | Low risk | Low risk | Unclear risk | Low risk | Low risk | NA | NA | Low risk | Low risk | NA |
| M-Kambafwile, 2017, primary care | Low risk | Low risk | Unclear risk | Low risk | Low risk | NA | NA | Low risk | Low risk | NA |
| Bates, 2013, hospital inpatients | High risk | Low risk | High risk | Low risk | Low risk | NA | NA | High risk | Low risk | NA |
| Gawa, 2011, hospital inpatients | Low risk | High risk | High risk | Unclear risk | High risk | NA | NA | High risk | High risk | NA |
| Hirao, 2007, hospital inpatients | Low risk | Low risk | High risk | Low risk | Low risk | NA | NA | Low risk | Low risk | NA |
| Jones-Lopez, 2014, inpatients | Low risk | Low risk | High risk | Low risk | Low risk | NA | NA | Low risk | Low risk | NA |
| Morse, 2008, hospital inpatients | Low risk | Low risk | High risk | Low risk | Low risk | Unclear risk | Unclear risk | Low risk | Low risk | Unclear risk |
| O'Grady, 2012, hospital inpatients | High risk | Low risk | High risk | Low risk | Low risk | NA | NA | High risk | Low risk | NA |
| Shah, 2009, hospital inpatients | Low risk | Low risk | High risk | Low risk | Low risk | Low risk | Unclear risk | Low risk | Low risk | Unclear risk |
| Talbot, 2004, hospital inpatients | Low risk | Low risk | High risk | Low risk | Low risk | NA | NA | Low risk | Low risk | NA |
| Yoon, 2012, hospital inpatients | Low risk | Low risk | High risk | Low risk | Low risk | Low risk | Low risk | Low risk | Low risk | Low risk |

*Only key domains are presented here a full excel table is available at the following link: <https://figshare.com/s/ead4d9c069268564d547> (Continued)

**Table S5** (Continued)

|  | **Outcome 1 - HIV testing** |  |  | **Outcome 2 -TB testing** |  | **Mortality Assessment** |  | **Overall risk of bias for outcomes** |  |  |
| --- | --- | --- | --- | --- | --- | --- | --- | --- | --- | --- |
| **Author and year, setting** | **Method of HIV testing** | **Uptake of HIV testing** | **Use of symptom screening questionnaire** | **Methods of diagnosis of TB** | **Participation rate of TB testing** | **Assessment of mortality** | **Adequacy of follow-up** | **HIV prevalence** | **TB prevalence** | **Mortality risk** |
| Nanta, 2011, mixed | Low risk | Low risk | High risk | Low risk | Unclear risk | NA | NA | Low risk | Unclear risk | NA |
| Okonkwo Chuks, 2013, mixed | Low risk | Low risk | High risk | Low risk | Unclear risk | NA | NA | Low risk | Unclear risk | NA |
| Rachow, 2011, mixed | Low risk | Low risk | High risk | Low risk | Low risk | NA | NA | Low risk | Low risk | NA |
| Van Lettow, 2014, mixed | High risk | High risk | High risk | Low risk | Low risk | Low risk | Low risk | High risk | Low risk | Low risk |
| Adjei, 2006, mixed | Low risk | Low risk | High risk | Low risk | Low risk | NA | NA | Low risk | Low risk | NA |
| Aliyu, 2013, mixed | Low risk | Low risk | High risk | Low risk | Low risk | NA | NA | Low risk | Low risk | NA |
| Kaur, 2011, mixed | Low risk | Low risk | High risk | Low risk | Low risk | NA | NA | Low risk | Low risk | NA |
| Boehme, Peru, 2011, mixed | Unclear risk | Unclear risk | High risk | Low risk | Low risk | NA | NA | Unclear risk | Low risk | NA |
| Legesse, 2010, mixed | Low risk | Low risk | High risk | Low risk | Low risk | NA | NA | Low risk | Low risk | NA |
| Pinyopornpanish, 2015, mixed | Low risk | Low risk | High risk | Low risk | Low risk | NA | NA | Low risk | Low risk | NA |
| Kawkitinarong, 2017, mixed | High risk | High risk | High risk | Low risk | Low risk | NA | NA | High risk | Low risk | NA |
| Mulder, 2017, mixed | High risk | High risk | High risk | Low risk | Low risk | NA | NA | High risk | Low risk | NA |
| Boehme, RSA, 2011 | High risk | High risk | High risk | Low risk | Low risk | NA | NA | High risk | Low risk | NA |
| Boehme, Uganda, 2011 | Low risk | Low risk | High risk | Low risk | Low risk | NA | NA | Low risk | Low risk | NA |
| Belay, 2015, mixed | Low risk | Low risk | High risk | Low risk | Low risk | NA | NA | Low risk | Low risk | NA |

*Only key domains are presented here a full excel table is available at the following link: <https://figshare.com/s/ead4d9c069268564d547>

| **Country,**  **author,**  **year ^a^** | **Setting** | **Participant Eligibility criteria** | **Participants with symptoms** | **Number screened for HIV** | **Number HIV positive** | **New HIV positive** | **Number needed to screen** |
| --- | --- | --- | --- | --- | --- | --- | --- |
|  |  |  | **N** | **n (%)** | **n (%)** |  |  |
| Malawi, Nliwasa, 2016 | Community | Cough ≥ 2 weeks | 178 | 162 (91.0) | 56/162 (34.6) | 15/162 (9.3) | 11 |
| Rwanda national TB prevalence survey, 2014 | Community | Cough (any duration) or abnormal CXR | 4,747 | 4,585 (96.6) | 218/4,585 (4.8) | 38/4,585 (0.83) | 121 |
| South Africa, Kranzer, 2012 | Community- mobile service | Any TB symptoms | 1,385 | 1,385 (100) | 758/1,385 (54.7) | 370/1,385 (26.7) | 4 |
| India, Achanta, 2012 | Primary care clinic | Cough ≥ 2 week or other symptoms | 2,918 | 2,465 (84.5) | 246/2,465 (10.0) | 84/2,465 (3.4) | 30 |
| Malawi, Nliwasa, 2016 | Primary care clinic | Cough ≥ 2 weeks | 273 | 251 (91.9) | 121/251 (48.2) | 87/251 (34.7) | 2 |
| Zambia, Mwansa-Kambafwile, 2017 | Primary care clinic | Any TB symptoms | 1,981 | 1,981 (100) | 1,442/1981 (72.8) | 516/1,981 (26.0) | 4 |
| Dlodlo, Zimbabwe, 2015 | Primary care clinic | Cough ≥ 2 weeks | 422 | 422 (100) | 297/422 (70.4) | 190/422 (42.0) | 2 |

1. **Number needed to screen to detect one new HIV positive case**
2. **Influence of study quality on HIV and TB estimates**

| **HIV prevalence** |  |  |  |  |  |
| --- | --- | --- | --- | --- | --- |
|  | **Studies** | **Participants**  **Screened** | **Median HIV prevalence** | **Univariate**  **meta-regression** |  |
|  |  |  | **% (IQR)** | **Prevalence ratio**  **(95% CI)** | **P** |
| Risk of bias |  |  |  |  |  |
| Low risk | 47 | 158,683 | 46.2 (20.3 – 69.7) | 1 |  |
| High risk | 15 | 25,918 | 34.0 (19.2 – 68.1) | 0.98 (0.81 - 1.19) | 0.865 |
| **TB prevalence** |  |  |  |  |  |
|  | **Studies** | **Participants**  **Screened** | **Median HIV prevalence** | **Univariate**  **meta-regression** |  |
|  |  |  | **% (IQR)** | **Prevalence ratio**  **(95% CI)** | **P** |
| Risk of bias |  |  |  |  |  |
| Low risk | 52 | 88,572 | 22.1 (11.1 – 39.8) | 1 |  |
| High risk | 7 | 66,595 | 15.4 (8.9 – 28.9) | 0.89 (0.74 – 1.08) | 0.744 |

1. **
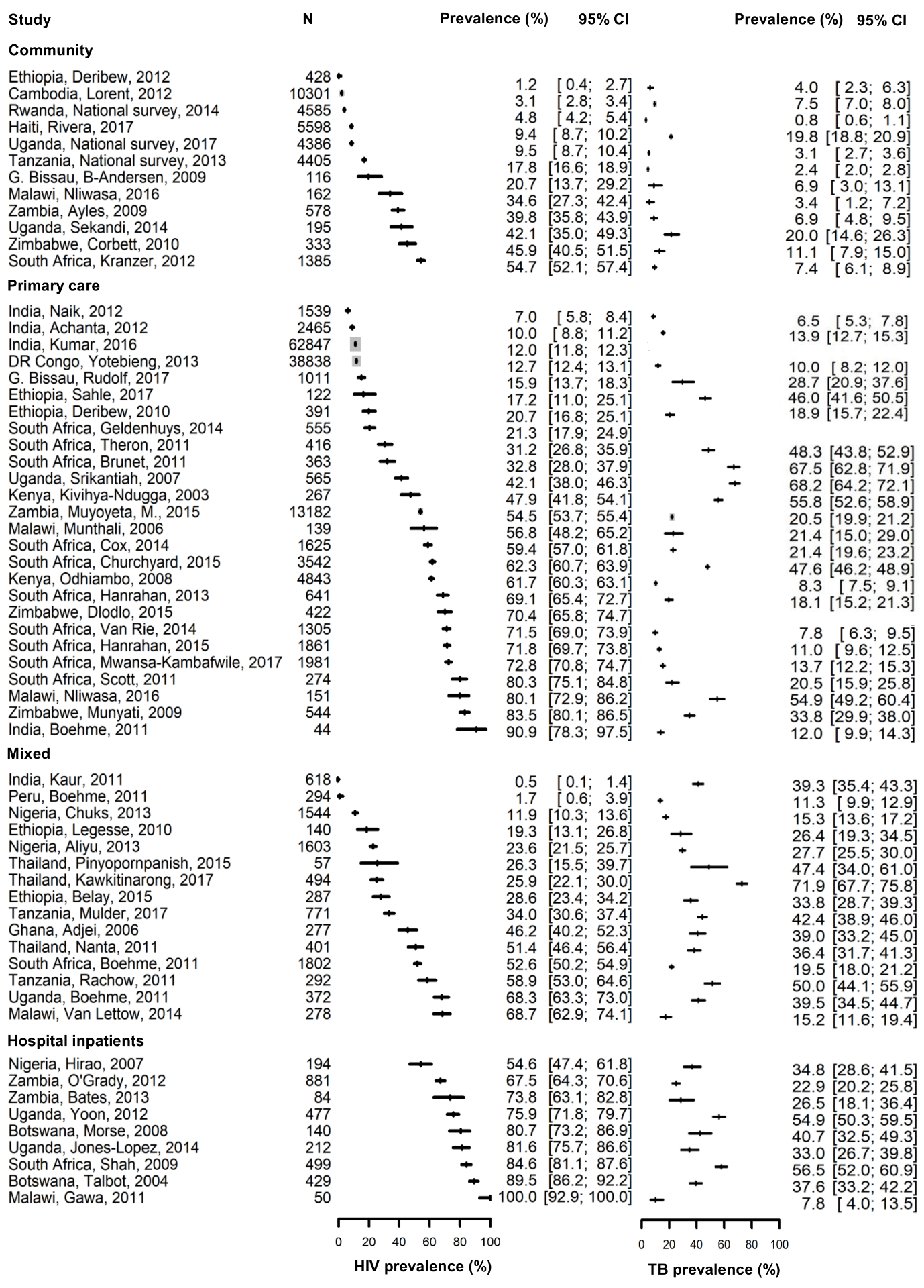
Forest plot of HIV and TB prevalence in adults with symptoms of TB stratified by level of health care**

Letter ‘N’ represents the number of participants with symptoms screened for HIV and TB


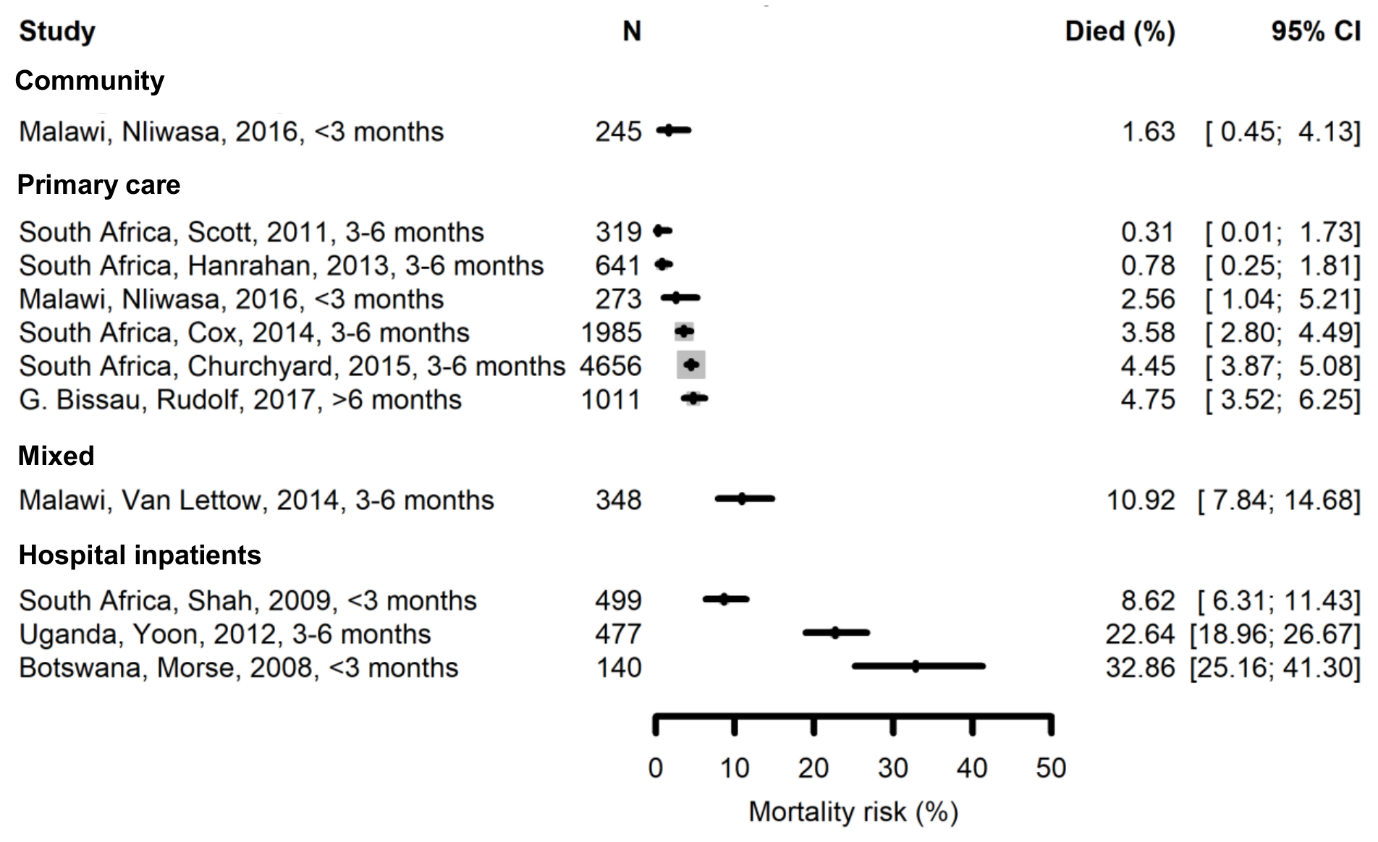


1. **Forest plot of mortality risk in adults with symptoms of TB stratified by level of care**

Letter ‘N’ represents the number of participants with symptoms

Mortality was assessed at maximum follow-up time of 6 months, specific periods are shown for each study

**References**

1. Wondimeneh Y, Muluye D, Belyhun Y. Prevalence of pulmonary tuberculosis and immunological profile of HIV co-infected patients in Northwest Ethiopia. *BMC Research Notes* 2012; **5**: 331.

2. Wesen A, Mitike G. Screening and case detection for tuberculosis among people living with HIV in Addis Ababa, Ethiopia. *Ethiopian Medical Journal* 2009; **47**(2): 109-15.

3. Were W, Moore D, Ekwaru P, et al. A simple screening tool for active tuberculosis in HIV-infected adults receiving antiretroviral treatment in Uganda. *International Journal of Tuberculosis & Lung Disease* 2009; **13**(1): 47-53.

4. Venkatesh PA, Bosch RJ, McIntosh K, Mugusi F, Msamanga G, Fawzi WW. Predictors of incident tuberculosis among HIV-1-infected women in Tanzania. *International Journal of Tuberculosis & Lung Disease* 2005; **9**(10): 1105-11.

5. van der Sande MA, Schim van der Loeff MF, Bennett RC, et al. Incidence of tuberculosis and survival after its diagnosis in patients infected with HIV-1 and HIV-2. *AIDS* 2004; **18**(14): 1933-41.

6. Tribble AC, Hamilton CD, Crump JA, et al. Missed opportunities for diagnosis of tuberculosis and human immunodeficiency virus co-infection in Moshi, Tanzania. *International Journal of Tuberculosis & Lung Disease* 2009; **13**(10): 1260-6.

7. Teck R, Ascurra O, Gomani P, et al. WHO clinical staging of HIV infection and disease, tuberculosis and eligibility for antiretroviral treatment: relationship to CD4 lymphocyte counts. *International Journal of Tuberculosis & Lung Disease* 2005; **9**(3): 258-62.

8. Sonnenberg P, Glynn JR, Fielding K, Murray J, Godfrey-Faussett P, Shearer S. How soon after infection with HIV does the risk of tuberculosis start to increase? A retrospective cohort study in South African gold miners. *Journal of Infectious Diseases* 2005; **191**(2): 150-8.

9. Shayo GA, Minja LT, Egwaga S, Bakari M, Mugusi FM. Symptom-based screening tool in ruling out active tuberculosis among HIV-infected patients eligible for isoniazid preventive therapy in Tanzania. *Tropical Medicine and International Health* 2014; **19**(6): 726-33.

10. Shah S, Demissie M, Lambert L, et al. Intensified tuberculosis case finding among HIV-Infected persons from a voluntary counseling and testing center in Addis Ababa, Ethiopia. *Journal of Acquired Immune Deficiency Syndromes: JAIDS* 2009; **50**(5): 537-45.

11. Shah NS, Anh MH, Thuy TT, et al. Population-based chest X-ray screening for pulmonary tuberculosis in people living with HIV/AIDS, An Giang, Vietnam. *International Journal of Tuberculosis & Lung Disease* 2008; **12**(4): 404-10.

12. Seyler C, Toure S, Messou E, Bonard D, Gabillard D, Anglaret X. Risk factors for active tuberculosis after antiretroviral treatment initiation in Abidjan. *American Journal of Respiratory & Critical Care Medicine* 2005; **172**(1): 123-7.

13. Pungrassami P, Kipp AM, Stewart PW, Chongsuvivatwong V, Strauss RP, Van Rie A. Tuberculosis and AIDS stigma among patients who delay seeking care for tuberculosis symptoms. *International Journal of Tuberculosis & Lung Disease* 2010; **14**(2): 181-7.

14. Peter J, Theron G, Chanda D, et al. Test characteristics and potential impact of the urine LAM lateral flow assay in HIV-infected outpatients under investigation for TB and able to self-expectorate sputum for diagnostic testing. *BMC Infectious Diseases* 2015; **15**(262).

15. Oni T, Burke R, Tsekela R, et al. High prevalence of subclinical tuberculosis in HIV-1-infected persons without advanced immunodeficiency: Implications for TB screening. *Thorax* 2011; **66**(8): 669-73.

16. Omair MA, Al-Ghamdi AA, Alrajhi AA. Incidence of tuberculosis in people living with the human immunodeficiency virus in Saudi Arabia. *International Journal of Tuberculosis & Lung Disease* 2010; **14**(5): 600-3.

17. Nunes EA, De Capitani EM, Coelho E, Panunto AC, Joaquim OA, Ramos Mde C. Mycobacterium tuberculosis and nontuberculous mycobacterial isolates among patients with recent HIV infection in Mozambique. *Jornal Brasileiro De Pneumologia: Publicacao Oficial Da Sociedade Brasileira De Pneumologia E Tisilogia* 2008; **34**(10): 822-8.

18. Ngowi BJ, Mfinanga SG, Bruun JN, Morkve O. Pulmonary tuberculosis among people living with HIV/AIDS attending care and treatment in rural northern Tanzania. *BMC Public Health* 2008; **8**: 341.

19. Mugisha B, Bock N, Mermin J, et al. Tuberculosis case finding and preventive therapy in an HIV voluntary counseling and testing center in Uganda. *International Journal of Tuberculosis & Lung Disease* 2006; **10**(7): 761-7.

20. Muga R, Ferreros I, Langohr K, et al. Changes in the incidence of tuberculosis in a cohort of HIV-seroconverters before and after the introduction of HAART. *AIDS* 2007; **21**(18): 2521-7.

21. Mtei L, Matee M, Herfort O, et al. High rates of clinical and subclinical tuberculosis among HIV-infected ambulatory subjects in Tanzania. *Clinical Infectious Diseases* 2005; **40**(10): 1500-7.

22. Moore D, Liechty C, Ekwaru P, et al. Prevalence, incidence and mortality associated with tuberculosis in HIV-infected patients initiating antiretroviral therapy in rural Uganda. *AIDS* 2007; **21**(6): 713-9.

23. Monkongdee P, McCarthy KD, Cain KP, et al. Yield of acid-fast smear and mycobacterial culture for tuberculosis diagnosis in people with human immunodeficiency virus. *American Journal of Respiratory & Critical Care Medicine* 2009; **180**(9): 903-8.

24. Mohammed A, Ehrlich R, Wood R, Cilliers F, Maartens G. Screening for tuberculosis in adults with advanced HIV infection prior to preventive therapy. *International Journal of Tuberculosis & Lung Disease* 2004; **8**(6): 792-5.

25. Meintjes G, Wilkinson RJ. Undiagnosed active tuberculosis in HIV-infected patients commencing antiretroviral therapy. *Clinical Infectious Diseases* 2010; **51**(7): 830-2.

26. Matee M, Mtei L, Lounasvaara T, et al. Sputum microscopy for the diagnosis of HIV-associated pulmonary tuberculosis in Tanzania. *BMC Public Health* 2008; **8**: 68.

27. Lawn SD, Kerkhoff AD, Vogt M, Wood R. Diagnostic accuracy of a low-cost, urine antigen, point-of-care screening assay for HIV-associated pulmonary tuberculosis before antiretroviral therapy: a descriptive study. *The Lancet Infectious Diseases* 2012; **12**(3): 201-9.

28. Kufa T, Mngomezulu V, Charalambous S, et al. Undiagnosed tuberculosis among HIV clinic attendees: association with antiretroviral therapy and implications for intensified case finding, isoniazid preventive therapy, and infection control. *Journal of Acquired Immune Deficiency Syndromes: JAIDS* 2012; **60**(2): e22-8.

29. Kranzer K, Zeinecker J, Ginsberg P, et al. Linkage to HIV care and antiretroviral therapy in Cape Town, South Africa. *PLoS ONE [Electronic Resource]* 2010; **5**(11): e13801.

30. Koole O, van Griensven J, Colebunders R. Tuberculosis screening and diagnosis in people with HIV. *New England Journal of Medicine* 2010; **362**(22): 2139; author reply -40.

31. J Peters MVDW, J Heunis, S Masuku, T Osoba. Barriers to implementing integrated TB-HIV service delivery in an antenatal care facility in Frances Baard District, Northern Cape, South Africa. 43rd World Conference on Lung Health of the International Union Against Tuberculosis and Lung Disease (The Union); 2012; Kuala Lumpur, Malaysia; 2012.

32. Hermans S, Nasuuna E, van Leth F, et al. Implementation and effect of intensified case finding on diagnosis of tuberculosis in a large urban HIV clinic in Uganda: a retrospective cohort study. *BMC Public Health* 2012; **12**: 674.

33. Hanifa Y, Fielding KL, Charalambous S, et al. Tuberculosis among adults starting antiretroviral therapy in South Africa: the need for routine case finding. *International Journal of Tuberculosis & Lung Disease* 2012; **16**(9): 1252-9.

34. Golub JE, Saraceni V, Cavalcante SC, et al. The impact of antiretroviral therapy and isoniazid preventive therapy on tuberculosis incidence in HIV-infected patients in Rio de Janeiro, Brazil. *AIDS* 2007; **21**(11): 1441-8.

35. Fabian J, Naicker S, Venter WD, et al. Urinary screening abnormalities in antiretroviral-naive HIV-infected outpatients and implications for management--a single-center study in South Africa. *Ethnicity & Disease* 2009; **19**(1 Suppl 1): S1-80-5.

36. Elden S, Lawes T, Kudsk-Iversen S, et al. Integrating intensified case finding of tuberculosis into HIV care: an evaluation from rural Swaziland. *BMC Health Services Research* 2011; **11**: 118.

37. Drain PK, Losina E, Coleman SM, et al. Diagnostic accuracy of a point-of-care urine test for tuberculosis screening among newly-diagnosed HIV-infected adults: a prospective, clinic-based study. *BMC Infectious Diseases* 2014; **14**: 110.

38. Drain PK, Losina E, Coleman SM, et al. Value of urine lipoarabinomannan grade and second test for optimizing clinic-based screening for HIV-associated pulmonary tuberculosis. *Journal of Acquired Immune Deficiency Syndromes: JAIDS* 2015; **68**(3): 274-80.

39. Day JH, Charalambous S, Fielding KL, Hayes RJ, Churchyard GJ, Grant AD. Screening for tuberculosis prior to isoniazid preventive therapy among HIV-infected gold miners in South Africa. *International Journal of Tuberculosis & Lung Disease* 2006; **10**(5): 523-9.

40. Chheng P, Tamhane A, Natpratan C, et al. Pulmonary tuberculosis among patients visiting a voluntary confidential counseling and testing center, Cambodia. *International Journal of Tuberculosis & Lung Disease* 2008; **12**(3 Suppl 1): 54-62.

41. Centers for Disease C, Prevention. Screening HIV-infected persons for tuberculosis--Cambodia, January 2004-February 2005. *MMWR - Morbidity & Mortality Weekly Report* 2005; **54**(46): 1177-80.

42. Cain KP, McCarthy KD, Heilig CM, et al. An algorithm for tuberculosis screening and diagnosis in people with HIV. *New England Journal of Medicine* 2010; **362**(8): 707-16.

43. Bonnet MM, Pinoges LL, Varaine FF, et al. Tuberculosis after HAART initiation in HIV-positive patients from five countries with a high tuberculosis burden. *AIDS* 2006; **20**(9): 1275-9.

44. Bhattacharya MK, Naik TN, Ghosh M, Jana S, Dutta P. Pulmonary tuberculosis among HIV seropositives attending a counseling center in Kolkata. *Indian Journal of Public Health* 2011; **55**(4): 329-31.

45. Batista J, de Albuquerque Mde F, Maruza M, et al. Incidence and risk factors for tuberculosis in people living with HIV: cohort from HIV referral health centers in Recife, Brazil. *PLoS ONE [Electronic Resource]* 2013; **8**(5): e63916.

46. Balcha TT, Sturegard E, Winqvist N, et al. Intensified tuberculosis case-finding in HIV-positive adults managed at Ethiopian health centers: diagnostic yield of Xpert MTB/RIF compared with smear microscopy and liquid culture. PLoS ONE [Electronic Resource]; 2014. p. e85478.

47. Albert H, Nakiyingi L, Sempa J, et al. Operational implementation of LED fluorescence microscopy in screening tuberculosis suspects in an urban HIV clinic in Uganda. *PLoS ONE [Electronic Resource]* 2013; **8**(9): e72556.

48. Ahmad Khan F, Verkuijl S, Parrish A, et al. Performance of symptom-based tuberculosis screening among people living with HIV: not as great as hoped. *AIDS* 2014; **28**(10): 1463-72.

49. Agizew TB, Arwady MA, Yoon JC, et al. Tuberculosis in asymptomatic HIV-infected adults with abnormal chest radiographs screened for tuberculosis prevention. *International Journal of Tuberculosis & Lung Disease* 2010; **14**(1): 45-51.

50. Agizew T, Bachhuber MA, Nyirenda S, et al. Association of chest radiographic abnormalities with tuberculosis disease in asymptomatic HIV-infected adults. *International Journal of Tuberculosis & Lung Disease* 2010; **14**(3): 324-31.

51. Worodria W, Massinga-Loembe M, Mazakpwe D, et al. Incidence and predictors of mortality and the effect of tuberculosis immune reconstitution inflammatory syndrome in a cohort of TB/HIV patients commencing antiretroviral therapy. *Journal of Acquired Immune Deficiency Syndromes: JAIDS* 2011; **58**(1): 32-7.

52. Tan CK, Hung CC, Lai CC, et al. Diagnosis of active tuberculosis by enzyme-linked immunospot assay for interferon-gamma in HIV-infected patients. *Journal of Acquired Immune Deficiency Syndromes: JAIDS* 2010; **53**(4): 546-7.

53. Talbot E, Munseri P, Teixeira P, et al. Test characteristics of urinary lipoarabinomannan and predictors of mortality among hospitalized HIV-infected tuberculosis suspects in Tanzania. *PLoS ONE [Electronic Resource]* 2012; **7**(3): e32876.

54. Swindells S, Komarow L, Tripathy S, et al. Screening for pulmonary tuberculosis in HIV-infected individuals: AIDS Clinical Trials Group Protocol A5253. *International Journal of Tuberculosis & Lung Disease* 2013; **17**(4): 532-9.

55. Swaminathan S, Paramasivan CN, Kumar SR, Mohan V, Venkatesan P. Unrecognised tuberculosis in HIV-infected patients: sputum culture is a useful tool. *International Journal of Tuberculosis & Lung Disease* 2004; **8**(7): 896-8.

56. Sudha M, Atul R. Prevalence of pulmonary tuberculosis in HIV positive individuals, its sensitivity and association with CD4 count. *International Journal of Current Microbiology and Applied Sciences* 2015; **4**(6): 292-304.

57. Shenoi SV, Brooks RP, Catterick K, Moll AP, Friedland GH. 'Cough officer' nurses in a general medical clinic successfully detect drug-susceptible and -resistant tuberculosis. *Public Health Action* 2013; **3**(1): 46-50.

58. Saranchuk P, Boulle A, Hilderbrand K, et al. Evaluation of a diagnostic algorithm for smear-negative pulmonary tuberculosis in HIV-infected adults. *South African Medical Journal Suid-Afrikaanse Tydskrif Vir Geneeskunde* 2007; **97**(7): 517-23.

59. Saldanha D, Gupta N, Shenoy S, Saralaya V. Prevalence of opportunistic infections in AIDS patients in Mangalore, Karnataka. *Tropical Doctor* 2008; **38**(3): 172-3.

60. Peter JG, Theron G, van Zyl-Smit R, et al. Diagnostic accuracy of a urine lipoarabinomannan strip-test for TB detection in HIV-infected hospitalised patients. *European Respiratory Journal* 2012; **40**(5): 1211-20.

61. Peter JG, Theron G, Muchinga TE, Govender U, Dheda K. The diagnostic accuracy of urine-based Xpert MTB/RIF in HIV-infected hospitalized patients who are smear-negative or sputum scarce. *PLoS ONE [Electronic Resource]* 2012; **7**(7): e39966.

62. Palanivel C, Kumar AMV, Mahalakshmi T, et al. Uptake of HIV testing and HIV positivity among presumptive tuberculosis patients at Puducherry, South India. *Public Health Action* 2013; **3**(3): 220-3.

63. Padmapriyadarsini C, Tripathy S, Sekar L, et al. Evaluation of a diagnostic algorithm for sputum smear-negative pulmonary tuberculosis in HIV-infected adults. *Journal of Acquired Immune Deficiency Syndromes: JAIDS* 2013; **63**(3): 331-8.

64. Oramasionwu GE, Heilig CM, Udomsantisuk N, et al. The utility of stool cultures for diagnosing tuberculosis in people living with the human immunodeficiency virus. *International Journal of Tuberculosis & Lung Disease* 2013; **17**(8): 1023-8.

65. Nakiyingi L, Moodley VM, Manabe YC, et al. Diagnostic accuracy of a rapid urine lipoarabinomannan test for tuberculosis in HIV-infected adults. *Journal of Acquired Immune Deficiency Syndromes: JAIDS* 2014; **66**(3): 270-9.

66. Nakatani SM, Messias-Reason IJ, Burger M, Cunha CA. Prevalence of Mycobacterium avium and Mycobacterium tuberculosis in blood cultures of Brazilian AIDS patients after introduction of highly active retroviral therapy. *Brazilian Journal of Infectious Diseases* 2005; **9**(6): 459-63.

67. Nakanjako D, Mayanja-Kizza H, Ouma J, et al. Tuberculosis and human immunodeficiency virus co-infections and their predictors at a hospital-based HIV/AIDS clinic in Uganda. *International Journal of Tuberculosis & Lung Disease* 2010; **14**(12): 1621-8.

68. McBride WJ, Greenhill AR. Human immunodeficiency virus and respiratory disorders: clinical and diagnostic considerations. *Papua New Guinea Medical Journal* 2010; **53**(3-4): 169-75.

69. Lawn SD, Kranzer K, Edwards DJ, McNally M, Bekker LG, Wood R. Tuberculosis during the first year of antiretroviral therapy in a South African cohort using an intensive pretreatment screening strategy. *Aids* 2010; **24**(9): 1323-8.

70. Lawn SD, Kerkhoff AD, Vogt M, Wood R. Diagnostic and prognostic value of serum C-reactive protein for screening for HIV-associated tuberculosis. *International Journal of Tuberculosis & Lung Disease* 2013; **17**(5): 636-43.

71. Lawn SD, Kerkhoff AD, Vogt M, Wood R. HIV-associated tuberculosis: relationship between disease severity and the sensitivity of new sputum-based and urine-based diagnostic assays. *BMC Medicine* 2013; **11**: 231.

72. Lawn SD, Kerkhoff AD, Vogt M, Wood R. High diagnostic yield of tuberculosis from screening urine samples from HIV-infected patients with advanced immunodeficiency using the Xpert MTB/RIF assay. *Journal of Acquired Immune Deficiency Syndromes: JAIDS* 2012; **60**(3): 289-94.

73. Lawn SD, Kerkhoff AD, Vogt M, Wood R. Clinical significance of lipoarabinomannan detection in urine using a low-cost point-of-care diagnostic assay for HIV-associated tuberculosis. *Aids* 2012; **26**(13): 1635-43.

74. Lawn SD, Kerkhoff AD, Vogt M, Ghebrekristos Y, Whitelaw A, Wood R. Characteristics and early outcomes of patients with Xpert MTB/RIF-negative pulmonary tuberculosis diagnosed during screening before antiretroviral therapy. *Clinical Infectious Diseases* 2012; **54**(8): 1071-9.

75. Lawn SD, Kerkhoff AD, Pahlana P, Vogt M, Wood R. Diagnostic yield of tuberculosis using sputum induction in HIV-positive patients before antiretroviral therapy. *International Journal of Tuberculosis & Lung Disease* 2012; **16**(10): 1354-7.

76. Lawn SD, Edwards DJ, Kranzer K, Vogt M, Bekker LG, Wood R. Urine lipoarabinomannan assay for tuberculosis screening before antiretroviral therapy diagnostic yield and association with immune reconstitution disease. *AIDS* 2009; **23**(14): 1875-80.

77. Lawn SD, Brooks SV, Kranzer K, et al. Screening for HIV-associated tuberculosis and rifampicin resistance before antiretroviral therapy using the Xpert MTB/RIF assay: a prospective study. *PLoS Medicine / Public Library of Science* 2011; **8**(7): e1001067.

78. Lawn SD, Badri M, Wood R. Tuberculosis among HIV-infected patients receiving HAART: long term incidence and risk factors in a South African cohort. *AIDS* 2005; **19**(18): 2109-16.

79. Kong BN, Harwell JI, Suos P, et al. Opportunistic infections and HIV clinical disease stage among patients presenting for care in Phnom Penh, Cambodia. *Southeast Asian Journal of Tropical Medicine & Public Health* 2007; **38**(1): 62-8.

80. Kisembo HN, Boon SD, Davis JL, et al. Chest radiographic findings of pulmonary tuberculosis in severely immunocompromised patients with the human immunodeficiency virus. *British Journal of Radiology* 2012; **85**(1014): e130-9.

81. Kim L, Heilig CM, McCarthy KD, et al. Symptom screen for identification of highly infectious tuberculosis in people living with HIV in Southeast Asia. *Journal of Acquired Immune Deficiency Syndromes: JAIDS* 2012; **60**(5): 519-24.

82. Kiertiburanakul S, Watcharatipagorn S, Chongtrakool P, Santanirand P. Epidemiology of bloodstream infections and predictive factors of mortality among HIV-infected adult patients in Thailand in the era of highly active antiretroviral therapy. *Japanese Journal of Infectious Diseases* 2012; **65**(1): 28-32.

83. Holtz TH, Kabera G, Mthiyane T, et al. Use of a WHO-recommended algorithm to reduce mortality in seriously ill patients with HIV infection and smear-negative pulmonary tuberculosis in South Africa: an observational cohort study. *The Lancet Infectious Diseases* 2011; **11**(7): 533-40.

84. Hermans SM, Kiragga AN, Schaefer P, Kambugu A, Hoepelman AI, Manabe YC. Incident tuberculosis during antiretroviral therapy contributes to suboptimal immune reconstitution in a large urban HIV clinic in sub-Saharan Africa. *PLoS ONE [Electronic Resource]* 2010; **5**(5): e10527.

85. Ha DT, Lan NT, Kiet VS, et al. Diagnosis of pulmonary tuberculosis in HIV-positive patients by microscopic observation drug susceptibility assay. *Journal of Clinical Microbiology* 2010; **48**(12): 4573-9.

86. Gutierrez EB, Gomes V, Picone CM, Suga H, Atomiya AN. Active tuberculosis and Mycobacterium tuberculosis latent infection in patients with HIV/AIDS. *HIV Medicine* 2009; **10**(9): 564-72.

87. Gounder CR, Kufa T, Wada NI, et al. Diagnostic accuracy of a urine lipoarabinomannan enzyme-linked immunosorbent assay for screening ambulatory HIV-infected persons for tuberculosis. *Journal of Acquired Immune Deficiency Syndromes: JAIDS* 2011; **58**(2): 219-23.

88. Feasey NA, Banada PP, Howson W, et al. Evaluation of Xpert MTB/RIF for detection of tuberculosis from blood samples of HIV-infected adults confirms Mycobacterium tuberculosis bacteremia as an indicator of poor prognosis. *Journal of Clinical Microbiology* 2013; **51**(7): 2311-6.

89. Dawson R, Masuka P, Edwards DJ, et al. Chest radiograph reading and recording system: evaluation for tuberculosis screening in patients with advanced HIV. *International Journal of Tuberculosis & Lung Disease* 2010; **14**(1): 52-8.

90. Davis JL, Worodria W, Kisembo H, et al. Clinical and radiographic factors do not accurately diagnose smear-negative tuberculosis in HIV-infected inpatients in Uganda: a cross-sectional study. *PLoS ONE [Electronic Resource]* 2010; **5**(3): e9859.

91. Conesa-Botella A, Loembe MM, Manabe YC, et al. Urinary lipoarabinomannan as predictor for the tuberculosis immune reconstitution inflammatory syndrome. *Journal of Acquired Immune Deficiency Syndromes: JAIDS* 2011; **58**(5): 463-8.

92. Cattamanchi A, Dowdy DW, Davis JL, et al. Sensitivity of direct versus concentrated sputum smear microscopy in HIV-infected patients suspected of having pulmonary tuberculosis. *BMC Infectious Diseases* 2009; **9**: 53.

93. Carriquiry G, Otero L, Gonzalez-Lagos E, et al. A diagnostic accuracy study of XpertMTB/RIF in HIV-positive patients with high clinical suspicion of pulmonary tuberculosis in Lima, Peru. *PLoS ONE [Electronic Resource]* 2012; **7**(9): e44626.

94. Bwana V, Tenu F, Magesa SM, Mfinanga SG. Smear positive pulmonary tuberculosis among HIV patients receiving highly active antiretroviral therapy in Dar es Salaam, Tanzania. *Tanzania journal of health research* 2011; **13**(1): 14-20.

95. Bedell RA, Anderson ST, van Lettow M, et al. High prevalence of tuberculosis and serious bloodstream infections in ambulatory individuals presenting for antiretroviral therapy in Malawi. *PLoS ONE [Electronic Resource]* 2012; **7**(6): e39347.

96. Bacha HA, Cimerman S, de Souza SA, Hadad DJ, Mendes CM. Prevalence of mycobacteremia in patients with AIDS and persistant fever. *Brazilian Journal of Infectious Diseases* 2004; **8**(4): 290-5.

97. Attili VS, Singh VP, Rai M, Varma DV, Sundar S. Evaluation of the status of tuberculosis as part of the clinical case definition of AIDS in India. *Postgraduate Medical Journal* 2005; **81**(956): 404-8.

98. Assefa D, Melaku Z, Gadissa T, Negash A, Hinderaker SG, Harries AD. Intensified tuberculosis case finding among people living with the human immunodeficiency virus in a hospital clinic in Ethiopia. *International Journal of Tuberculosis & Lung Disease* 2011; **15**(3): 411-3.

99. Andama AO, Boon Sd, Meya D, et al. Prevalence and outcomes of cryptococcal antigenemia in HIV-seropositive patients hospitalized for suspected tuberculosis in Uganda. *JAIDS, Journal of Acquired Immune Deficiency Syndromes* 2013; **63**(2): 189-94.

100. Affolabi D, Akpona R, Odoun M, et al. Smear-negative, culture-positive pulmonary tuberculosis among patients with chronic cough in Cotonou, Benin. *International Journal of Tuberculosis and Lung Disease* 2011; **15**(1): 67-70.

101. Akanbi MO, Achenbach C, Taiwo B, et al. Evaluation of gene xpert for routine diagnosis of HIV-associated tuberculosis in Nigeria: A prospective cohort study. *BMC Pulmonary Medicine* 2017; **17 (1) (no pagination)**(87).

102. Lessells RJ, Cooke GS, McGrath N, Nicol MP, Newell ML, Godfrey-Faussett P. Impact of Point-of-Care Xpert MTB/RIF on Tuberculosis Treatment Initiation. A Cluster-randomized Trial. *American Journal of Respiratory & Critical Care Medicine* 2017; **196**(7): 901-10.

103. Alamo ST, Kunutsor S, Walley J, et al. Performance of the new WHO diagnostic algorithm for smear-negative pulmonary tuberculosis in HIV prevalent settings: a multisite study in Uganda. *Tropical Medicine & International Health* 2012; **17**(7): 884-95.

104. Breen RA, Hardy GA, Perrin FM, et al. Rapid diagnosis of smear-negative tuberculosis using immunology and microbiology with induced sputum in HIV-infected and uninfected individuals. *PLoS ONE [Electronic Resource]* 2007; **2**(12): e1335.

105. Davis JL, Huang L, Worodria W, et al. Nucleic acid amplification tests for diagnosis of smear-negative TB in a high HIV-prevalence setting: a prospective cohort study. *PLoS ONE [Electronic Resource]* 2011; **6**(1): e16321.

106. Desta K, Asrat D, Lemma E, Gebeyehu M, Feleke B. Prevalence of smear negative pulmonary tuberculosis among patients visiting St. Peter's Tuberculosis Specialized Hospital, Addis Ababa, Ethiopia. *Ethiopian Medical Journal* 2009; **47**(1): 17-24.

107. Dimairo M, MacPherson P, Bandason T, et al. The risk and timing of tuberculosis diagnosed in smear-negative TB suspects: a 12 month cohort study in Harare, Zimbabwe. *PLoS ONE [Electronic Resource]* 2010; **5**(7): e11849.

108. Huerga H, Varaine F, Okwaro E, et al. Performance of the 2007 WHO algorithm to diagnose smear-negative pulmonary tuberculosis in a HIV prevalent setting. *PLoS ONE [Electronic Resource]* 2012; **7**(12): e51336.

109. Macpherson P, Dimairo M, Bandason T, et al. Risk factors for mortality in smear-negative tuberculosis suspects: a cohort study in Harare, Zimbabwe. *International Journal of Tuberculosis & Lung Disease* 2011; **15**(10): 1390-6.

110. Porskrog A, Bjerregaard-Andersen M, Oliveira I, et al. Enhanced tuberculosis identification through 1-month follow-up of smear-negative tuberculosis suspects. *International Journal of Tuberculosis & Lung Disease* 2011; **15**(4): 459-64.

111. Rabna P, Andersen A, Wejse C, et al. High mortality risk among individuals assumed to be TB-negative can be predicted using a simple test. *Tropical Medicine & International Health* 2009; **14**(9): 986-94.

112. Siddiqi K, Walley J, Khan MA, Shah K, Safdar N. Clinical guidelines to diagnose smear-negative pulmonary tuberculosis in Pakistan, a country with low-HIV prevalence. *Tropical Medicine & International Health* 2006; **11**(3): 323-31.

113. Somashekar N, Chadha VK, Praseeja P, et al. Role of pre-Xpert screening using chest X-ray in early diagnosis of smear-negative pulmonary tuberculosis. *International Journal of Tuberculosis & Lung Disease* 2014; **18**(10): 1243-4.

114. Theron G, Peter J, Meldau R, et al. Accuracy and impact of Xpert MTB/RIF for the diagnosis of smear-negative or sputum-scarce tuberculosis using bronchoalveolar lavage fluid. *Thorax* 2013; **68**(11): 1043-51.

115. Wekesa C, Kirenga BJ, Joloba ML, Bwanga F, Katamba A, Kamya MR. Chest X-ray vs. Xpert MTB/RIF assay for the diagnosis of sputum smear-negative tuberculosis in Uganda. *International Journal of Tuberculosis & Lung Disease* 2014; **18**(2): 216-9.

116. Wilson D, Badri M, Maartens G. Performance of serum C-reactive protein as a screening test for smear-negative tuberculosis in an ambulatory high HIV prevalence population. *PLoS ONE [Electronic Resource]* 2011; **6**(1): e15248.

117. Wilson D, Mbhele L, Badri M, et al. Evaluation of the World Health Organization algorithm for the diagnosis of HIV-associated sputum smear-negative tuberculosis. *International Journal of Tuberculosis & Lung Disease* 2011; **15**(7): 919-24.

118. Woldesemayat EM, Datiko DG, Lindtjorn B. Follow-up of chronic coughers improves tuberculosis case finding: results from a community-based cohort study in southern Ethiopia. *PLoS One* 2015; **10**(2): e0116324.

119. Badal-Faesen S, Firnhaber C, Kendall MA, et al. Impact of larger sputum volume on xpert MTB/RIF assay detection of mycobacterium tuberculosis in smear-negative individuals with suspected tuberculosis. *Journal of Clinical Medicine* 2017; **6 (8) (no pagination)**(78).

120. E Fair CM, L Samu, M Hudson, G Chamie, S G Mfinanga, P Hopewell. Acceptability of home based HIV testing during household tuberculosis contact investigation in Dar es Salaam, Tanzania. 45th World Conference on Lung Health of the International Union Against Tuberculosis and Lung Disease (The Union); 2014; Barcelona, Spain; 2014.

121. Guwatudde D, Nakakeeto M, Jones-Lopez EC, et al. Tuberculosis in household contacts of infectious cases in Kampala, Uganda. *American Journal of Epidemiology* 2003; **158**(9): 887-98.

122. Hill PC, Jackson-Sillah DJ, Fox A, et al. Incidence of tuberculosis and the predictive value of ELISPOT and Mantoux tests in Gambian case contacts. *PLoS ONE [Electronic Resource]* 2008; **3**(1): e1379.

123. J Galea CCCM, R Calderon, S Shin, R Lobaton, M Becerra, L Lecca Garcia, M Murray. Feasibility and acceptability of in-home, rapid HIV testing among contacts of tuberculosis patients in Lima, Peru. 43rd World Conference on Lung Health of the International Union Against Tuberculosis and Lung Disease (The Union); 2012; Kuala Lumpur, Malaysia; 2012.

124. Li J, Marks SM, Driver CR, et al. Human immunodeficiency virus counseling, testing, and referral of close contacts to patients with pulmonary tuberculosis: feasibility and costs. *Journal of Public Health Management & Practice* 2007; **13**(3): 252-62.

125. LoBue PA, LeClair JJ, Moser KS. Contact investigation for cases of pulmonary Mycobacterium bovis. *International Journal of Tuberculosis & Lung Disease* 2004; **8**(7): 868-72.

126. Mwansa-Kambafwile J, McCarthy K, Gharbaharan V, Venter FW, Maitshotlo B, Black A. Tuberculosis case finding: evaluation of a paper slip method to trace contacts. *PLoS ONE [Electronic Resource]* 2013; **8**(9): e75757.

127. Suggaravetsiri P, Yanai H, Chongsuvivatwong V, Naimpasan O, Akarasewi P. Integrated counseling and screening for tuberculosis and HIV among household contacts of tuberculosis patients in an endemic area of HIV infection: Chiang Rai, Thailand. *International Journal of Tuberculosis & Lung Disease* 2003; **7**(12 Suppl 3): S424-31.

128. van Schalkwyk C, Variava E, Shapiro AE, et al. Incidence of TB and HIV in prospectively followed household contacts of TB index patients in South Africa. *PLoS ONE [Electronic Resource]* 2014; **9**(4): e95372.

129. Zachariah R, Spielmann MP, Harries AD, et al. Passive versus active tuberculosis case finding and isoniazid preventive therapy among household contacts in a rural district of Malawi. *International Journal of Tuberculosis & Lung Disease* 2003; **7**(11): 1033-9.

130. Kranzer K, Bekker LG, van Schaik N, et al. Community health care workers in South Africa are at increased risk for tuberculosis. *South African Medical Journal Suid-Afrikaanse Tydskrif Vir Geneeskunde* 2010; **100**(4): 224, 6.

131. Kassa-Kelembho E, Mbolidi CD, Service YB, Morvan J, Minssart P. Bacteremia in adults admitted to the Department of Medicine of Bangui Community Hospital (Central African Republic). *Acta Tropica* 2003; **89**(1): 67-72.

132. Gupta A, Mbwambo J, Mteza I, et al. Active case finding for tuberculosis among people who inject drugs on methadone treatment in Dar es Salaam, Tanzania. *International Journal of Tuberculosis & Lung Disease* 2014; **18**(7): 793-8.

133. A M Venkatachalaiah DG, B Naik, K G Deepak, S Shastri, S Srinath, P Dewan, A D Harries. Case fatality among HIV-infected tuberculosis patients with CD4 count > 350 cells/mm^3^. 43rd World Conference on Lung Health of the International Union Against Tuberculosis and Lung Disease (The Union); 2012; Kuala Lumpur, Malaysia; 2012.

134. Kali PB, Gray GE, Violari A, Chaisson RE, McIntyre JA, Martinson NA. Combining PMTCT with active case finding for tuberculosis. *Journal of Acquired Immune Deficiency Syndromes: JAIDS* 2006; **42**(3): 379-81.

135. Abed Al-Darraji HA, Abd Razak H, Ng KP, Altice FL, Kamarulzaman A. The diagnostic performance of a single GeneXpert MTB/RIF assay in an intensified tuberculosis case finding survey among HIV-infected prisoners in Malaysia. *PLoS ONE [Electronic Resource]* 2013; **8**(9): e73717.

136. Ndwiga C, Birungi H, Undie CC, Weyenga H, Sitienei J. Feasibility and effect of integrating tuberculosis screening and detection in postnatal care services: an operations research study. *BMC Health Services Research* 2013; **13**: 99.

137. Hoffmann CJ, Variava E, Rakgokong M, et al. High prevalence of pulmonary tuberculosis but low sensitivity of symptom screening among HIV-infected pregnant women in South Africa. *PLoS ONE* 2013; **8**(4).

138. Lewis JJ, Charalambous S, Day JH, et al. HIV infection does not affect active case finding of tuberculosis in South African gold miners. *American Journal of Respiratory & Critical Care Medicine* 2009; **180**(12): 1271-8.

139. Corbett EL, Charalambous S, Moloi VM, et al. Human immunodeficiency virus and the prevalence of undiagnosed tuberculosis in African gold miners. *American Journal of Respiratory & Critical Care Medicine* 2004; **170**(6): 673-9.

140. Park HH, Girdler-Brown BV, Churchyard GJ, White NW, Ehrlich RI. Incidence of tuberculosis and HIV and progression of silicosis and lung function impairment among former Basotho gold miners. *American Journal of Industrial Medicine* 2009; **52**(12): 901-8.

141. Soto A, Solari L, Gotuzzo E, Acinelli R, Vargas D, Van der Stuyft P. Performance of an algorithm based on WHO recommendations for the diagnosis of smear-negative pulmonary tuberculosis in patients without HIV infection. *Tropical Medicine & International Health* 2011; **16**(4): 424-30.

142. Gupta A, Nayak U, Ram M, et al. Postpartum tuberculosis incidence and mortality among HIV-infected women and their infants in Pune, India, 2002-2005. *Clinical Infectious Diseases* 2007; **45**(2): 241-9.

143. Moges B, Amare B, Asfaw F, et al. Prevalence of smear positive pulmonary tuberculosis among prisoners in North Gondar Zone Prison, northwest Ethiopia. *BMC Infectious Diseases* 2012; **12**: 352.

144. Margolis B, Al-Darraji HA, Wickersham JA, Kamarulzaman A, Altice FL. Prevalence of tuberculosis symptoms and latent tuberculous infection among prisoners in northeastern Malaysia. *International Journal of Tuberculosis & Lung Disease* 2013; **17**(12): 1538-44.

145. Kazi AM, Shah SA, Jenkins CA, Shepherd BE, Vermund SH. Risk factors and prevalence of tuberculosis, human immunodeficiency virus, syphilis, hepatitis B virus, and hepatitis C virus among prisoners in Pakistan. *International Journal of Infectious Diseases* 2010; **14 Suppl 3**: e60-6.

146. Meints L, Chescheir N. Screening for infectious diseases in pregnant, foreign-born women from multiple global areas. *Journal of Reproductive Medicine* 2010; **55**(9-10): 382-6.

147. Churchyard GJ, Fielding KL, Lewis JJ, Chihota VN, Hanifa Y, Grant AD. Symptom and chest radiographic screening for infectious tuberculosis prior to starting isoniazid preventive therapy: yield and proportion missed at screening. *AIDS* 2010; **24 Suppl 5**: S19-27.

148. Kosgei RJ, Ndavi PM, Ong'ech JO, et al. Symptom screen: diagnostic usefulness in detecting pulmonary tuberculosis in HIV-infected pregnant women in Kenya. *Public Health Action* 2011; **1**(2): 30-3.

149. Gupta A, Chandrasekhar A, Gupte N, et al. Symptom screening among HIV-infected pregnant women is acceptable and has high negative predictive value for active tuberculosis. *Clinical Infectious Diseases* 2011; **53**(10): 1015-8.

150. Modi S, Cavanaugh JS, Shiraishi RW, et al. Symptom-Based Screening for Tuberculosis Among Pregnant Women Living With HIV in Kenya. *Topics in Antiviral Medicine* 2014; **22 (e-1)**: 422-3.

151. Nachega J, Coetzee J, Adendorff T, et al. Tuberculosis active case-finding in a mother-to-child HIV transmission prevention programme in Soweto, South Africa. *AIDS* 2003; **17**(9): 1398-400.

152. Churchyard GJ, Fielding K, Roux S, et al. Twelve-monthly versus six-monthly radiological screening for active case-finding of tuberculosis: a randomised controlled trial. *Thorax* 2011; **66**(2): 134-9.

153. Bates M, Ahmed Y, Chilukutu L, et al. Use of the Xpert() MTB/RIF assay for diagnosing pulmonary tuberculosis comorbidity and multidrug-resistant TB in obstetrics and gynaecology inpatient wards at the University Teaching Hospital, Lusaka, Zambia. *Tropical Medicine & International Health* 2013; **18**(9): 1134-40.

154. Cohen T, Murray M, Wallengren K, Alvarez GG, Samuel EY, Wilson D. The prevalence and drug sensitivity of tuberculosis among patients dying in hospital in KwaZulu-Natal, South Africa: a postmortem study. *PLoS Medicine / Public Library of Science* 2010; **7**(6): e1000296.

155. Suwanpimolkul G, Kawkitinarong K, Manosuthi W, et al. Utility of urine lipoarabinomannan (LAM) in diagnosing tuberculosis and predicting mortality with and without HIV: prospective TB cohort from the Thailand Big City TB Research Network. *International Journal of Infectious Diseases* 2017; **59**: 96-102.

156. Calligaro GL, Zijenah LS, Peter JG, et al. Effect of new tuberculosis diagnostic technologies on community-based intensified case finding: a multicentre randomised controlled trial. *The Lancet Infectious Diseases* 2017; **17**(4): 441-50.

157. Jerene D, Hiruy N, Jemal I, et al. The yield and feasibility of integrated screening for TB, diabetes and HIV in four public hospitals in Ethiopia. *International Health* 2017; **9**(2): 100-4.

158. Masenga SK, Mubila H, Hamooya BM. Rifampicin resistance in mycobacterium tuberculosis patients using GeneXpert at Livingstone Central Hospital for the year 2015: A cross sectional explorative study. *BMC Infectious Diseases* 2017; **17 (1) (no pagination)**(640).

159. Albert H, Manabe Y, Lukyamuzi G, et al. Performance of three LED-based fluorescence microscopy systems for detection of tuberculosis in Uganda. *PLoS ONE [Electronic Resource]* 2010; **5**(12): e15206.

160. Bassett IV, Giddy J, Chaisson CE, et al. A randomized trial to optimize HIV/TB care in South Africa: design of the Sizanani trial. *BMC Infectious Diseases* 2013; **13**: 390.

161. Belay M, Bjune G, Abebe F. Prevalence of tuberculosis, HIV, and TB-HIV co-infection among pulmonary tuberculosis suspects in a predominantly pastoralist area, northeast Ethiopia. *Global health action* 2015; **8**: 27949.

162. Boehme C, Molokova E, Minja F, et al. Detection of mycobacterial lipoarabinomannan with an antigen-capture ELISA in unprocessed urine of Tanzanian patients with suspected tuberculosis. *Transactions of the Royal Society of Tropical Medicine & Hygiene* 2005; **99**(12): 893-900.

163. Boehme CC, Nabeta P, Hillemann D, et al. Rapid molecular detection of tuberculosis and rifampin resistance. *New England Journal of Medicine* 2010; **363**(11): 1005-15.

164. Bonnet M, Gagnidze L, Githui W, et al. Performance of LED-based fluorescence microscopy to diagnose tuberculosis in a peripheral health centre in Nairobi. *PLoS ONE [Electronic Resource]* 2011; **6**(2): e17214.

165. Cambanis A, Ramsay A, Wirkom V, Tata E, Cuevas LE. Investing time in microscopy: an opportunity to optimise smear-based case detection of tuberculosis. *International Journal of Tuberculosis & Lung Disease* 2007; **11**(1): 40-5.

166. Cattamanchi A, Davis JL, Worodria W, et al. Sensitivity and specificity of fluorescence microscopy for diagnosing pulmonary tuberculosis in a high HIV prevalence setting. *International Journal of Tuberculosis & Lung Disease* 2009; **13**(9): 1130-6.

167. Chaidir L, Parwati I, Annisa J, et al. Implementation of LED fluorescence microscopy for diagnosis of pulmonary and HIV-associated tuberculosis in a hospital setting in Indonesia. *PLoS ONE [Electronic Resource]* 2013; **8**(4): e61727.

168. Cleeff MRAv, Kivihya-Ndugga LE, Meme H, Odhiambo JA, Klatser PR. The role and performance of chest X-ray for the diagnosis of tuberculosis: a cost-effectiveness analysis in Nairobi, Kenya. *BMC Infectious Diseases* 2005; **5**(111).

169. Corbett EL, Bandason T, Duong T, et al. Comparison of two active case-finding strategies for community-based diagnosis of symptomatic smear-positive tuberculosis and control of infectious tuberculosis in Harare, Zimbabwe (DETECTB): a cluster-randomised trial. *Lancet* 2010; **376**(9748): 1244-53.

170. Desalu OO, Oluwafemi JA, Ojo O. Respiratory diseases morbidity and mortality among adults attending a tertiary hospital in Nigeria. *Jornal Brasileiro De Pneumologia: Publicacao Oficial Da Sociedade Brasileira De Pneumologia E Tisilogia* 2009; **35**(8): 745-52.

171. Gasana M, Vandebriel G, Kabanda G, et al. Integrating tuberculosis and HIV care in rural Rwanda. *International Journal of Tuberculosis & Lung Disease* 2008; **12**(3 Suppl 1): 39-43.

172. Heidebrecht CL, Podewils LJ, Pym AS, Cohen T, Mthiyane T, Wilson D. Assessing the utility of Xpert((R)) MTB/RIF as a screening tool for patients admitted to medical wards in South Africa. *Scientific reports* 2016; **6**: 19391.

173. Kapata N, Chanda-Kapata P, Ngosa W, et al. The Prevalence of Tuberculosis in Zambia: Results from the First National TB Prevalence Survey, 2013-2014. *PLoS One* 2016; **11**(1): e0146392.

174. Kunimoto D, Long R. Tuberculosis: still overlooked as a cause of community-acquired pneumonia--how not to miss it. *Respiratory Care Clinics of North America* 2005; **11**(1): 25-34.

175. Lockman S, Hone N, Kenyon TA, et al. Etiology of pulmonary infections in predominantly HIV-infected adults with suspected tuberculosis, Botswana. *International Journal of Tuberculosis & Lung Disease* 2003; **7**(8): 714-23.

176. Yotebieng M LW, E Basaki, M Batumbula, M Tabala, E Mungoyo, R Mangala, W Behets. PITC of tuberculosis suspects and prevalence of HIV among tuberculosis suspects in Kinshasa and Kisangani, Democratic Republic of Congo. 44rd World Conference on Lung Health of the International Union Against Tuberculosis and Lung Disease (The Union); 2013; Paris, France; 2013.

177. Meintjes G, Schoeman H, Morroni C, Wilson D, Maartens G. Patient and provider delay in tuberculosis suspects from communities with a high HIV prevalence in South Africa: a cross-sectional study. *BMC Infectious Diseases* 2008; **8**: 72.

178. Noeske J, Dopico E, Torrea G, Wang H, Van Deun A. Two vs. three sputum samples for microscopic detection of tuberculosis in a high HIV prevalence population. *International Journal of Tuberculosis & Lung Disease* 2009; **13**(7): 842-7.

179. Nyamande K, Lalloo UG, John M. TB presenting as community-acquired pneumonia in a setting of high TB incidence and high HIV prevalence. *International Journal of Tuberculosis & Lung Disease* 2007; **11**(12): 1308-13.

180. Page AL, Ardizzoni E, Lassovsky M, et al. Routine use of Xpert(R) MTB/RIF in areas with different prevalences of HIV and drug-resistant tuberculosis. *The international journal of tuberculosis and lung disease : the official journal of the International Union against Tuberculosis and Lung Disease* 2015; **19**(9): 1078-83, i-iii.

181. Sebhatu M, Kiflom B, Seyoum M, et al. Determining the burden of tuberculosis in Eritrea: a new approach. *Bull World Health Organ* 2007; **85**(8): 593-9.

182. Srisuwanvilai LO, Monkongdee P, Podewils LJ, et al. Performance of the BACTEC MGIT 960 compared with solid media for detection of Mycobacterium in Bangkok, Thailand. *Diagnostic Microbiology & Infectious Disease* 2008; **61**(4): 402-7.

183. Talbot EA, Hone NM, Moffat HJ, et al. The validity of HIV testing using sputum from suspected tuberculosis patients, Botswana, 2001. *International Journal of Tuberculosis & Lung Disease* 2003; **7**(8): 710-3.

184. Tan CK, Lai CC, Liao CH, et al. Mycobacterial bacteraemia in patients infected and not infected with human immunodeficiency virus, Taiwan. *Clinical Microbiology & Infection* 2010; **16**(6): 627-30.

185. Torrea G, Van de Perre P, Ouedraogo M, et al. PCR-based detection of the Mycobacterium tuberculosis complex in urine of HIV-infected and uninfected pulmonary and extrapulmonary tuberculosis patients in Burkina Faso. *Journal of Medical Microbiology* 2005; **54**(Pt 1): 39-44.

186. Van Rie A, Patel MR, Nana M, et al. Integration and task shifting for TB/HIV care and treatment in highly resource-scarce settings: one size may not fit all. *Journal of Acquired Immune Deficiency Syndromes: JAIDS* 2014; **65**(3): e110-7.

187. Green C, Huggett JF, Talbot E, Mwaba P, Reither K, Zumla AI. Rapid diagnosis of tuberculosis through the detection of mycobacterial DNA in urine by nucleic acid amplification methods. *The Lancet Infectious Diseases* 2009; **9**(8): 505-11.

188. Devi SB, Naorem S, Jeetenkumar Singh T, Singh Ksh B, Prasad L, Devi Th S. HIV and TB co-infection (A study from RIMS Hospital, Manipur). *Journal, Indian Academy of Clinical Medicine* 2005; **6**(3): 220-3.

189. Lawson L, Yassin MA, Thacher TD, et al. Clinical presentation of adults with pulmonary tuberculosis with and without HIV infection in Nigeria. *Scandinavian Journal of Infectious Diseases* 2008; **40**(1): 30-5.

190. Mutetwa R, Boehme C, Dimairo M, et al. Diagnostic accuracy of commercial urinary lipoarabinomannan detection in African tuberculosis suspects and patients. *International Journal of Tuberculosis & Lung Disease* 2009; **13**(10): 1253-9.

191. Peters RP, Zijlstra EE, Schijffelen MJ, et al. A prospective study of bloodstream infections as cause of fever in Malawi: clinical predictors and implications for management. *Tropical Medicine & International Health* 2004; **9**(8): 928-34.

192. Wood R, Middelkoop K, Myer L, et al. Undiagnosed tuberculosis in a community with high HIV prevalence: implications for tuberculosis control. *American Journal of Respiratory & Critical Care Medicine* 2007; **175**(1): 87-93.

193. Ayles H, Muyoyeta M, Du Toit E, et al. Effect of household and community interventions on the burden of tuberculosis in southern Africa: the ZAMSTAR community-randomised trial. *Lancet* 2013; **382**(9899): 1183-94.

194. Gelalcha AG, Kebede A, Mamo H. Light-emitting diode fluorescent microscopy and Xpert MTB/RIF assay for diagnosis of pulmonary tuberculosis among patients attending Ambo hospital, west-central Ethiopia. *BMC Infectious Diseases* 2017; **17 (1) (no pagination)**(613).

195. Jaleta KN, Gizachew M, Gelaw B, Tesfa H, Getaneh A, Biadgo B. Rifampicin-resistant Mycobacterium tuberculosis among tuberculosis-presumptive cases at University of Gondar hospital, northwest Ethiopia. *Infection and Drug Resistance* 2017; **10**: 185-92.

196. Mavenyengwa RT, Shaduka E, Maposa I. Evaluation of the Xpert MTB/RIF assay and microscopy for the diagnosis of Mycobacterium tuberculosis in Namibia. *Infectious Diseases of Poverty* 2017; **6**(1): 13.

197. Claassens M, van Schalkwyk C, den Haan L, et al. High prevalence of tuberculosis and insufficient case detection in two communities in the Western Cape, South Africa. *PLoS ONE [Electronic Resource]* 2013; **8**(4): e58689.

198. Claassens MM, Jacobs E, Cyster E, et al. Tuberculosis cases missed in primary health care facilities: should we redefine case finding? *International Journal of Tuberculosis & Lung Disease* 2013; **17**(5): 608-14.

199. Shapiro AE, Variava E, Rakgokong MH, et al. Community-based targeted case finding for tuberculosis and HIV in household contacts of patients with tuberculosis in South Africa. *American Journal of Respiratory & Critical Care Medicine* 2012; **185**(10): 1110-6.

200. Shrivastava SR, Shrivastava PS. Tuberculosis: active case finding survey in an urban area of India, in 2012. *Journal of research in health sciences* 2012; **13**(1): 19-23.

201. Agizew T, Boyd R, Ndwapi N, et al. Peripheral clinic versus centralized laboratory-based Xpert MTB/RIF performance: Experience gained from a pragmatic, stepped-wedge trial in Botswana. *PLoS ONE [Electronic Resource]* 2017; **12**(8): e0183237.

202. Bello G, Faragher B, Sanudi L, et al. The effect of engaging unpaid informal providers on case detection and treatment initiation rates for TB and HIV in rural Malawi (Triage Plus): A cluster randomised health system intervention trial. *PLoS ONE [Electronic Resource]* 2017; **12**(9): e0183312.

203. Ochang E, Emanghe U, Ewa A, et al. Evaluation of pulmonary tuberculosis case detection improvement with the deployment of XpertMTB/Rif in the tuberculosis control program of cross River State, Nigeria. *International Journal of Mycobacteriology* 2017; **6**(1): 94-6.

204. Ohene SA, Bonsu F, Hanson-Nortey NN, et al. Provider initiated tuberculosis case finding in outpatient departments of health care facilities in Ghana: Yield by screening strategy and target group. *BMC Infectious Diseases* 2017; **17 (1) (no pagination)**(739).

205. Oshi D, Omeje J, Oshi S, et al. An evaluation of innovative community-based approaches and systematic tuberculosis screening to improve tuberculosis case detection in Ebonyi State, Nigeria. *International Journal of Mycobacteriology* 2017; **6**(3): 246-52.

206. Reddy S, Ntoyanto S, Sakadavan Y, et al. Detecting Mycobacterium tuberculosis using the loop-mediated isothermal amplification test in South Africa. *International Journal of Tuberculosis and Lung Disease* 2017; **21**(10): 1154-60.

207. Getahun H, Raviglione M. Active case-finding for TB in the community: time to act. *Lancet* 2010; **376**(9748): 1205-6.

208. Mavhu W, Dauya E, Bandason T, et al. Chronic cough and its association with TB-HIV co-infection: factors affecting help-seeking behaviour in Harare, Zimbabwe. *Tropical Medicine & International Health* 2010; **15**(5): 574-9.

209. Poudel A, Pandey BD, Lekhak B, Rijal B, Sapkota BR, Suzuki Y. Clinical profiling and use of loop-mediated isothermal amplification assay for rapid detection of Mycobacterium tuberculosis from sputum. *Kathmandu University Medical Journal* 2009; **7**(26): 109-14.

210. Chartier L, Leng C, Sire JM, et al. Factors associated with negative direct sputum examination in Asian and African HIV-infected patients with tuberculosis (ANRS 1260). *PLoS ONE [Electronic Resource]* 2011; **6**(6): e21212.

211. Wolday D, Hailu B, Girma M, Hailu E, Sanders E, Fontanet AL. Low CD4+ T-cell count and high HIV viral load precede the development of tuberculosis disease in a cohort of HIV-positive Ethiopians. *Ethiopian Medical Journal* 2003; **41 Suppl 1**: 67-73.

212. Vassall A, Seme A, Compernolle P, Meheus F. Patient costs of accessing collaborative tuberculosis and human immunodeficiency virus interventions in Ethiopia. *International Journal of Tuberculosis & Lung Disease* 2010; **14**(5): 604-10.

213. van't Hoog AH, Meme HK, Laserson KF, et al. Screening strategies for tuberculosis prevalence surveys: the value of chest radiography and symptoms. *PLoS ONE [Electronic Resource]* 2012; **7**(7): e38691.

214. Cleeff Mv, Kivihya-Ndugga L, Githui W, et al. Cost-effectiveness of polymerase chain reaction versus Ziehl-Neelsen smear microscopy for diagnosis of tuberculosis in Kenya. *International Journal of Tuberculosis and Lung Disease* 2005; **9**(8): 877-83.

215. K Fielding SG, K McCarthy, E Sinanovic, M Nicol, L D Mametja, W Stevens, G Churchyard. High self-reported HIV positive status among clinic attendees suspected of tuberculosis enrolled in a cluster randomised trial of Xpert® MTB/RIF, South Africa. 44th World Conference on Lung Health of the International Union Against Tuberculosis and Lung Disease (The Union). Paris, France; 2013.

216. Corbett EL, Bandason T, Cheung YB, et al. Epidemiology of tuberculosis in a high HIV prevalence population provided with enhanced diagnosis of symptomatic disease. *PLoS Medicine / Public Library of Science* 2007; **4**(1): e22.

217. Munseri PJ, Bakari M, Pallangyo K, Sandstrom E. Tuberculosis in HIV voluntary counselling and testing centres in Dar es Salaam, Tanzania. *Scandinavian Journal of Infectious Diseases* 2010; **42**(10): 767-74.

218. Corbett EL, Bandason T, Cheung YB, et al. Prevalent infectious tuberculosis in Harare, Zimbabwe: burden, risk factors and implications for control. *International Journal of Tuberculosis & Lung Disease* 2009; **13**(10): 1231-7.

219. Bello G, Faragher B, Sanudi L, et al. Engaging informal health care providers in case detection for tuberculosis and HIV in rural Malawi. 44th World Conference on Lung Health of the International Union Against Tuberculosis and Lung Disease (The Union); 2013; Paris, France; 2013.

220. Bazile J, Makungwa H, Wang R, et al. Household chart, a useful tool for improving tuberculosis and HIV case fi nding among other medical conditions at the community level in rural Malawi. 43rd World Conference on Lung Health of the International Union Against Tuberculosis and Lung Disease (The Union); 2012; Kuala Lumpur, Malaysia; 2012.

221. Lawson L, Lawson JO, Olajide I, et al. Sex differences in the clinical presentation of urban Nigerian patients with pulmonary tuberculosis. *West African Journal of Medicine* 2008; **27**(2): 82-6.

222. Machao G, Lekone P, Lere T, et al. Linkage to TB treatment in Botswana among Tebelopele clients who screened positive for TB, 2008 - 2012. 8th IAS Conference on HIV Pathogenesis, Treatment and Prevention 19 - 22 July 2015; 2014; Vancouver, Canada; 2014.

223. Naidoo P, Du Toit E, Dunbar R, Caldwell J, Beyers N, Enarson DA. Does the introduction of the Xpert® MTB/RIF test result in an increased tuberculosis diagnostic yield in a routine operational setting in Cape Town? 43rd World Conference on Lung Health of the International Union Against Tuberculosis and Lung Disease (The Union); 2012; Kuala Lumpur, Malaysia; 2012.

224. Hamilton C, Reddy E, Lancaster K, et al. High mortality in patients with TB symptoms in Moshi, Tanzania: Considerations for TB diagnostics, lung health and health systems strengthening. *American Journal of Respiratory and Critical Care Medicine* 2010; **181 (1 MeetingAbstracts)**.

225. Ngadaya ES, Kimaro GD, Sandi E, et al. Performance of xpert MTB/RIF assay for pulmonary tuberculosis and rifampicin resistance under clinical settings in Tanzania, a low income high tuberculosis burden country. *American Journal of Respiratory and Critical Care Medicine Conference: American Thoracic Society International Conference, ATS* 2017; **195**(no pagination).

226. Sarin R, Vohra V, Khalid UK, Sharma PP, Chadha V, Sharada MA. Prevalence of pulmonary tuberculosis among adults in selected slums of Delhi city. *Indian Journal of Tuberculosis* 2017.

227. Vargas D, Garcia L, Gilman RH, et al. Diagnosis of sputum-scarce HIV-associated pulmonary tuberculosis in Lima, Peru. *Lancet* 2005; **365**(9454): 150-2.

228. Malekmohammad M, Marjani M, Tabarsi P, et al. Diagnostic yield of post-bronchoscopy sputum smear in pulmonary tuberculosis. *Scandinavian Journal of Infectious Diseases* 2012; **44**(5): 369-73.

229. Nandagopal B, Sankar S, Lingesan K, Appu KC, Sridharan G, Gopinathan AK. Evaluation of a nested PCR targeting IS6110 of Mycobacterium tuberculosis for detection of the organism in the leukocyte fraction of blood samples. *Indian Journal of Medical Microbiology* 2010; **28**(3): 227-32.

230. Kibiki GS, Mulder B, van der Ven AJ, et al. Laboratory diagnosis of pulmonary tuberculosis in TB and HIV endemic settings and the contribution of real time PCR for M. tuberculosis in bronchoalveolar lavage fluid. *Tropical Medicine & International Health* 2007; **12**(10): 1210-7.
